# Gating the electron transfer at a monocopper centre through the supramolecular coordination of water molecules within a protein chamber mimic†
†Electronic supplementary information (ESI) available: General procedures, details on electrochemical experiments and simulations, ^1^H NMR data, mass analysis, computational studies, and X-ray data. See DOI: 10.1039/c8sc03124j


**DOI:** 10.1039/c8sc03124j

**Published:** 2018-08-30

**Authors:** Nicolas Le Poul, Benoit Colasson, Grégory Thiabaud, Dany Jeanne Dit Fouque, Claudio Iacobucci, Antony Memboeuf, Bénédicte Douziech, Jan Řezáč, Thierry Prangé, Aurélien de la Lande, Olivia Reinaud, Yves Le Mest

**Affiliations:** a Laboratoire de Chimie , Electrochimie Moléculaires et Chimie Analytique , UMR CNRS 6521 , Université de Brest , 29238 Brest , France . Email: yves.lemest@univ-brest.fr ; Email: nicolas.lepoul@univ-brest.fr ; Email: dany.jeanneditfouque@univ-brest.fr ; Email: iacobucci.claudio@gmail.com ; Email: antony.memboeuf@univ-brest.fr ; Email: benedicte.douziech@univ-brest.fr; b Laboratoire de Chimie et Biochimie Pharmacologiques et Toxicologiques , UMR CNRS 8601 , Université Paris Descartes , 75006 Paris , France . Email: olivia.reinaud@parisdescartes.fr ; Email: benoit.colasson@parisdescartes.fr; c Institute of Organic Chemistry and Biochemistry , Academy of Sciences of the Czech Republic , Flemingovonám. 2 , 166 10 Prague 6 , Czech Republic . Email: rezac@uochb.cas.cz; d Laboratoire de Cristallographie et de Résonance Magnétique Nucléaire , Biologiques (CNRS UMR 8015) , Université Paris Descartes , 4, Avenue de l’Observatoire , 75006 Paris , France . Email: thierry.prange@parisdescartes.fr; e Laboratoire de Chimie Physique , UMR CNRS 8000 , Université Paris Sud , 91405 Orsay , France . Email: aurelien.de-la-lande@u-psud.fr

## Abstract

Functionality of enzymes is strongly related to water dynamic processes. The control of the redox potential for metallo-enzymes is intimately linked to the mediation of water molecules in the first and second coordination spheres. Here, we report a unique example of supramolecular control of the redox properties of a biomimetic monocopper complex by water molecules. It is shown that the copper complex based on a calix[6]arene covalently capped with a tetradentate [tris(2-methylpyridyl)amine] (tmpa) core, embedding the metal ion in a hydrophobic cavity, can exist in three different states. The first system displays a totally irreversible redox behaviour. It corresponds to the reduction of the 5-coordinate mono-aqua-Cu^II^ complex, which is the thermodynamic species in the +II state. The second system is detected at a high redox potential. It is ascribed to an “empty cavity” or “water-free” state, where the Cu^I^ ion sits in a 4-coordinate trigonal environment provided by the tmpa cap. This complex is the thermodynamic species in the +I state under “dry conditions”. Surprisingly, a third redox system appears as the water concentration is increased. Under water-saturation conditions, it displays a pseudo-reversible behaviour at a low scan rate at the mid-point from the water-free and aqua species. This third system is not observed with the Cu-tmpa complex deprived of a cavity. In the calix[6]cavity environment, it is ascribed to a species where a pair of water molecules is hosted by the calixarene cavity. A molecular mechanism for the Cu^II^/Cu^I^ redox process with an interplay of (H_2_O)_*x*_ (*x* = 0, 1, 2) hosting is proposed on the basis of computational studies. Such an unusual behaviour is ascribed to the unexpected stabilization of the Cu^I^ state by inclusion of the pair of water molecules. This phenomenon strongly evidences the drastic influence of the interaction between water molecules and a hydrophobic cavity on controlling the thermodynamics and kinetics of the Cu^II^/Cu^I^ electron transfer process.

## Abstract 2

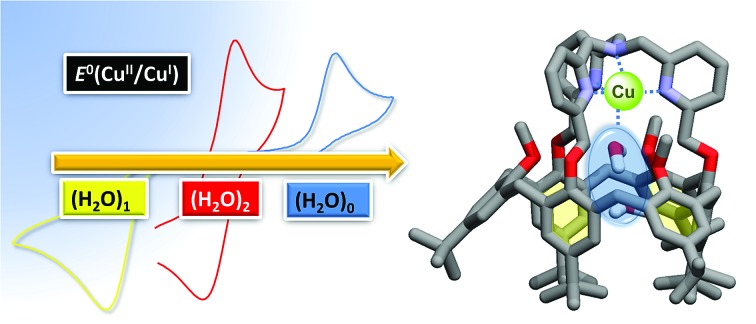
Functionality of enzymes is strongly related to water dynamic processes.

## Introduction

The role of water in biochemical events has been for a long time neglected, water being considered only for its solvation properties. Recently, more attention has been paid to its involvement in a large number of biological processes.^1^,^2^ It is now widely accepted that water has a crucial effect on both the structure^1^,^3^ and functionality of proteins (folding,^4^–^7^ molecular recognition,^8^ electron transfer,^9^–^11^ proton delivery^12^ and catalysis^13^,^14^). Its action covers a wide range of localizations, from cavities and distal pockets^15^,^16^ to inter-protein spaces.^17^ Water has been recognized as a particularly good mediator for transferring electrons between remote biological redox cofactors by quantum tunneling,^10^,^18^,^19^ thanks to the existence of multiple strong hydrogen bonding channels favoured by a constrained environment.^20^ For metallo-enzymes requiring dioxygen binding, it has been well recognized that water hosting in the hydrophobic pocket defining the active site plays key roles in the catalytic activity and control. For example, water release is a pre-requirement for the reduction of the resting Fe^III^ state in P-450 enzymes, and consequently for dioxygen binding.^21^ It has also been demonstrated that a pair of water molecules plays a key role in O–O bond heterolytic cleavage leading to the active species.^22^ More recently, it has been proposed that embedment of a water-cluster in the active site of cytochrome c-oxidase has a strong impact on its redox activity.^14^,^16^ Water release from the metal centre is also a necessary step for α-ketoglutarate-dependent non-heme mono-oxygenases. In copper enzymes, water molecules are suspected to play key roles in their catalytic cycles as well. For instance, a rare example of a bis-aqua adduct was recently reported for a Cu^II^ site of a Cu nitrite reductase (RpNiR).^23^ Water molecules were shown to specifically protect it from nitrite coordination at Cu^II^ and favour catalytic activation at Cu^I^ by displacement of the water ligand. In copper mono-oxygenases, water access or gating in the active site pocket may be key for generating a Cu^I^ state prone to O_2_ activation. This may also be implied in the fast reduction of the Cu^II^ state at the end of the catalytic cycle to regenerate the Cu^I^ active species. So far, the influence of water on the thermodynamics of redox biological systems has been little investigated.^24^ Most of the recent studies related to this subject have been devoted to the modification of the second coordination sphere in metalloproteins (*i.e.* peptidic residues) with the aim of tuning the redox properties and functionalities.^25^–^27^

**Fig. 1:**
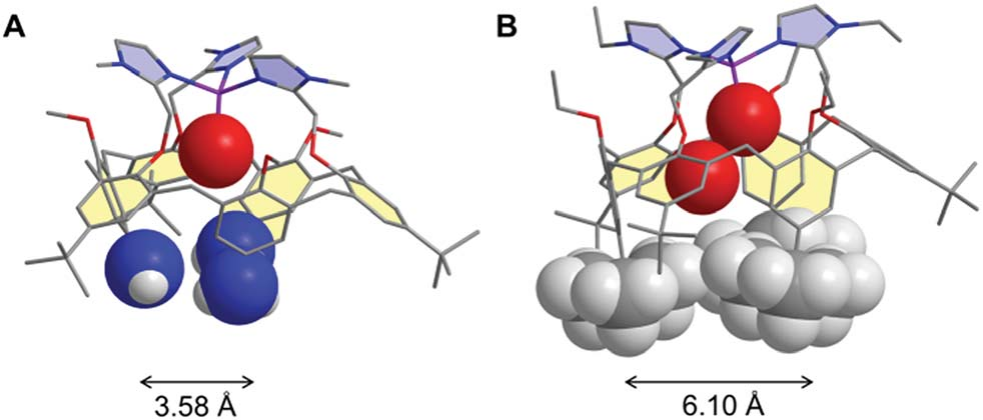
Comparison of the XRD structures of the tris-amino, tris-*t*Bu (A) and hexa-*t*Bu (B) calixarene-based Zn^II^–aqua complexes with a space-filling representation of the guest water molecules and the large rim inner substituents evidencing a different cavity space. Hydrogen atoms and counter anions have been omitted for clarity.

On the chemistry side, several examples of supramolecular objects such as organic cages or cavities able to encapsulate one or several water molecules have been described.^28^–^32^ Such systems are based on the ability of water molecules to form hydrogen-bonded clusters, hence neutralizing their intrinsic high polarity. Only few studies in solution have reported the encapsulation of a definite number of water molecules.^33^,^34^ Remarkably, for one of them, the water molecule was trapped in apolar C_60_ under high-pressure conditions.^33^ However, the electrochemical properties of the fullerene were not modified by the inner H_2_O molecule, probably due to the absence of electronic host/guest coupling. Another interesting redox-inactive example was obtained with Zn^II^ complexes based on calix[6]arenes. According to the substitution pattern at the large rim of the calixarene ligand, the number of water molecules inside the funnel could be varied. As shown in Fig. 1, only one H_2_O molecule coordinated to the metal centre was trapped when three amino and three *t*Bu groups were present at the large rim of the calixarene, whereas two water molecules were identified for the complex-bearing six *t*Bu moieties (Fig. 1).^28^,^29^

It is noteworthy that the trapping of a pair of water molecules inside the calix[6]cone was established in the solid state (XRD analysis) as well as in solution (by ^1^H NMR spectroscopy in CDCl_3_). As previously discussed,^29^ such a difference between these two complexes is due to the substitution at the large rim: for the tris-amino derivative, the OH–π interaction between the bent aniline unit and the guest water molecule shrinks the cavity size, thus adapting it to the smallness of the guest ligand (the average distance between the nitrogen atoms is 3.58 Å, Fig. 1A). For the hexa-*t*Bu complex, the bulky *t*Bu groups define a larger cavity space (the average distance between the central *t*Bu carbon atoms is 6.10 Å, Fig. 1B) hence allowing the inclusion of a second guest water molecule which is in interaction with the first one *via* a strong hydrogen bond.

Being interested in cavity effects associated with biomimetic redox centres, we thought of exploring the redox properties of a redox-active metal ion embedded in the calix[6]arene cone, knowing that the latter defines an open space prone for hosting a well-defined number of water molecules.

**Fig. 2:**
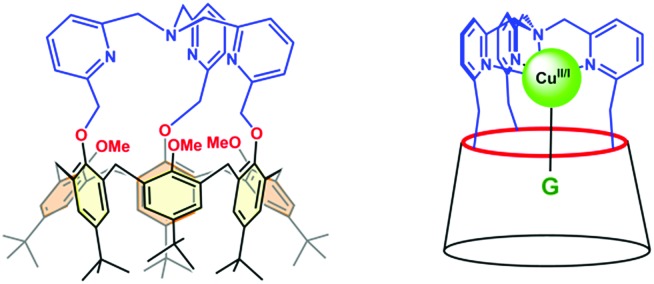
Schematic of the calix[6]tmpa ligand and the corresponding host–guest (G) Cu complex.

Among the different systems that we have described up to now,^35^ our choice was a calix[6]azacryptand featuring a [tris(2-methylpyridyl)amine] (tmpa) cap (Fig. 2). Indeed, a recent study by some of us concerning water coordination associated with the Cu^II^/Cu^I^ electron transfer within the tmpa ligand showed decoordination of the water molecule upon monoelectronic reduction of the Cu^II^ complex. Moreover, back-coordination of the water ligand in the Cu^I^ state was detected when increasing the water content and/or decreasing the timescale of the experiment.^36^ In the present study, we focus on water inclusion inside the hydrophobic calix[6]arene cavity and its effects on the redox properties of the copper centre bound to the tmpa core covalently attached to the macrocyclic conic scaffold.

## Results and discussion

### Description of the calix[6]tmpa copper system

The structure of the calix[6]tmpa ligand is displayed in Fig. 2. The corresponding Cu^II^ and Cu^I^ complexes were synthesized as previously reported^37^,^38^ by reacting one equiv. of metal salts, Cu^II^(OH_2_)_6_(ClO_4_)_2_ and Cu^I^(CF_3_SO_3_), respectively, in a non-coordinating solvent. In this system, the tmpa unit closes the small rim of the cone-shape cavity, precluding coordination to ligands in the *exo* position. The metal ion is embedded at the top of the half-open cavity that acts as a funnel controlling guest ligand exchange. Importantly, the small rim of the calixarene, which defines the second coordination sphere of the embedded metal ion, prevents simultaneous coordination of two guest ligands, thus leaving a single coordination site accessible to a ligand.^39^ The cavity can open and close (breathing), and adapt to the size of the entrapped guest (induced-fit).^29^

**Fig. 3:**
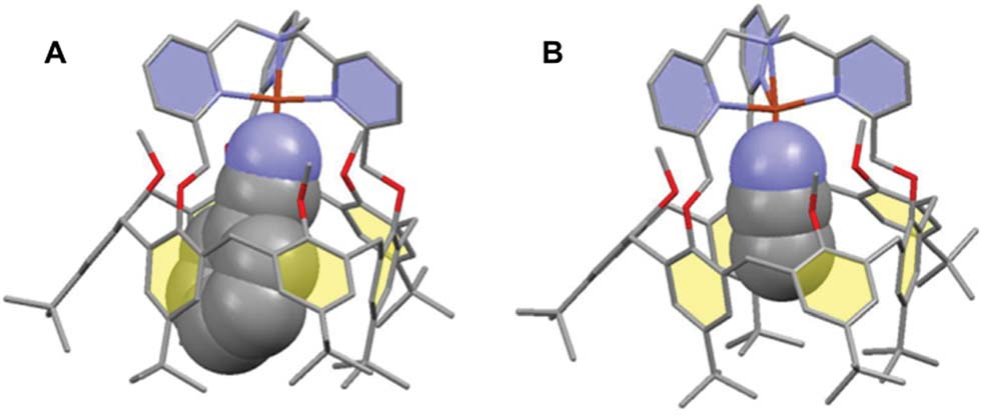
XRD structures of two Cu^II^ complexes based on the calix[6]tmpa scaffold. The guest ligand bound in the calixarene cavity: (A) PhCN and (B) MeCN. In both cases, the metal centre sits in a strongly axial trigonal bipyramidal environment (see ref. 37 and the ESI†) (hydrogen atoms and counterions are omitted for clarity).

Such supramolecular features mimic the hydrophobic pocket in Cu enzymes. In the presence of coordinating molecules, the Cu complexes readily form host–guest adducts, provided the guest can fit into the calixarene cavity. As illustrated in Fig. 3 displaying the XRD structures of the two Cu^II^ nitrilo complexes, this cavity can host guest ligands of different sizes without dramatic modifications (see ESI†). In chloroform or dichloromethane, and in the strict absence of any trace of the coordinating solvent, these copper complexes were identified as a monocationic “empty cavity”^40^ 4-coordinate species, [Cu^I^(calix[6]tmpa)]^+^ in the +I oxidation state,^38^ and a dicationic 5-coordinate mono-aqua complex, [Cu^II^(calix[6]tmpa)(H_2_O)]^2+^ in the +II oxidation state.^37^ Previous electrochemical studies by cyclic voltammetry (CV) showed that these Cu complexes display an irreversible redox behaviour due to the interconversion between a water-free Cu^I^ species and a mono-aqua Cu^II^ species.^41^ The aqua ligand is readily exchanged for small neutral donors such as MeCN and DMF to give rise to the corresponding host–guest dicationic Cu^II^ complexes that display reversible redox behaviours. Very interestingly, CV studies evidenced, during the electron exchange at the +II state, the kinetic trapping of a transient species, different from the thermodynamic species. This very unusual phenomenon is a direct consequence of the control exerted by the calixarene cavity. The latter, imposing a dissociative process at the Cu^II^ state, precludes the more favoured associative pathway and blocks the guest interconversion at the CV time scale. This study thus highlighted the crucial role that embedment of a reactive redox metal ion in a funnel-like cavity has in ligand exchange associated with electron transfer. We now report another impressive phenomenon related to the water content in the cavity of the Cu^I^ and Cu^II^ funnel complexes. It highlights the key role water molecules have in the control of the redox potential of the embedded copper ion and thus, possibly, in the redox activity of electron transfer metalloproteins and enzymes.

### Studies under dry conditions

**Fig. 4:**
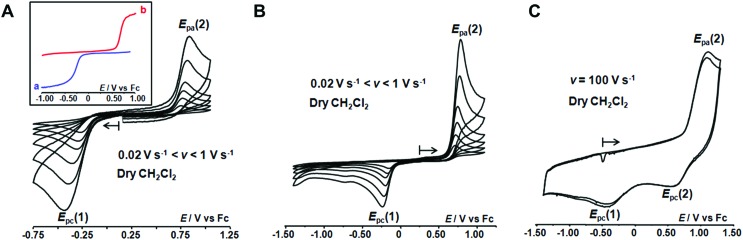
CVs at a Pt electrode in anhydrous CH_2_Cl_2_/NBu_4_PF_6_ (0.1 M) at [complex] = 10^–3^ M: (A) [Cu^II^(calix[6]tmpa)(H_2_O)]^2+^ and (B) [Cu^I^(calix[6]tmpa)]^+^ at varying scan rates, 0.02 V s^–1^ < *v* < 1 V s^–1^. Inset in (A): RDEV (a) before and (b) after electrolysis of [Cu^II^(calix[6]tmpa)(H_2_O)]^2+^ at –0.50 V *vs.* Fc. (C) CV at *v* = 100 V s^–1^ of [Cu^I^(calix[6]tmpa)]^+^ (2 cycles).

The redox behaviour of the aqua Cu^II^ complex, [Cu^II^(calix[6]tmpa)(H_2_O)]^2+^, was first investigated under dry and inert conditions in CH_2_Cl_2_.^42^ An irreversible cathodic peak at *E*_pc_(1) = –0.35 V *vs.* Fc (*E*^0^_(Fc+/Fc)_ = +0.64 V *vs.* NHE under the present conditions), associated with an irreversible anodic peak at *E*_pa_(2) = +0.75 V, was observed at 0.02 < *v* < 5 V s^–1^ by CV (Fig. 4A). Rotating disk electrode voltammetry (RDEV) before and after electrolysis (*n* = 1 e^–^) showed the cathodic and anodic waves, respectively, corresponding to both CV peaks (inset, Fig. 4A). A counter electrolysis restored the initial RDEV cathodic wave. This observation revealed a chemically reversible process as a square scheme mechanism with two interchangeable species stable at different redox potentials. Indeed, the CV in CH_2_Cl_2_ of the synthesized Cu^I^ complex displays irreversible peaks at *E*_pa_(2) = +0.75 V and *E*_pc_(1) = –0.20 V (Fig. 4B). The reverse oxidation peak at *E*_pa_(1) could not be detected even at high scan rates (Fig. 4C and S1†). Conversely, the reverse peak *E*_pc_(2) was detected at a high scan rate (100 V s^–1^) while *i*_pc_(1) decreased (Fig. 4C). In the second scan, the oxidation peak at *E*_pa_(1) was not observed. In agreement with the spectroscopic data of the Cu^II^ and Cu^I^-(calix[6]tmpa) complexes,^37^,^38^ and analogous with previous electrochemical studies with the related calix[6]tren-Cu system,^43^ the peak at *E*_pc_(1) is ascribed to the reduction of the aqua Cu^II^ complex (H_2_O coordinated to Cu^II^ inside the calixarene cone), whereas the anodic peak at *E*_pa_(2) corresponds to the oxidation of the water-free Cu^I^ complex (no H_2_O coordination to Cu^I^).^37^

**Scheme 1:**
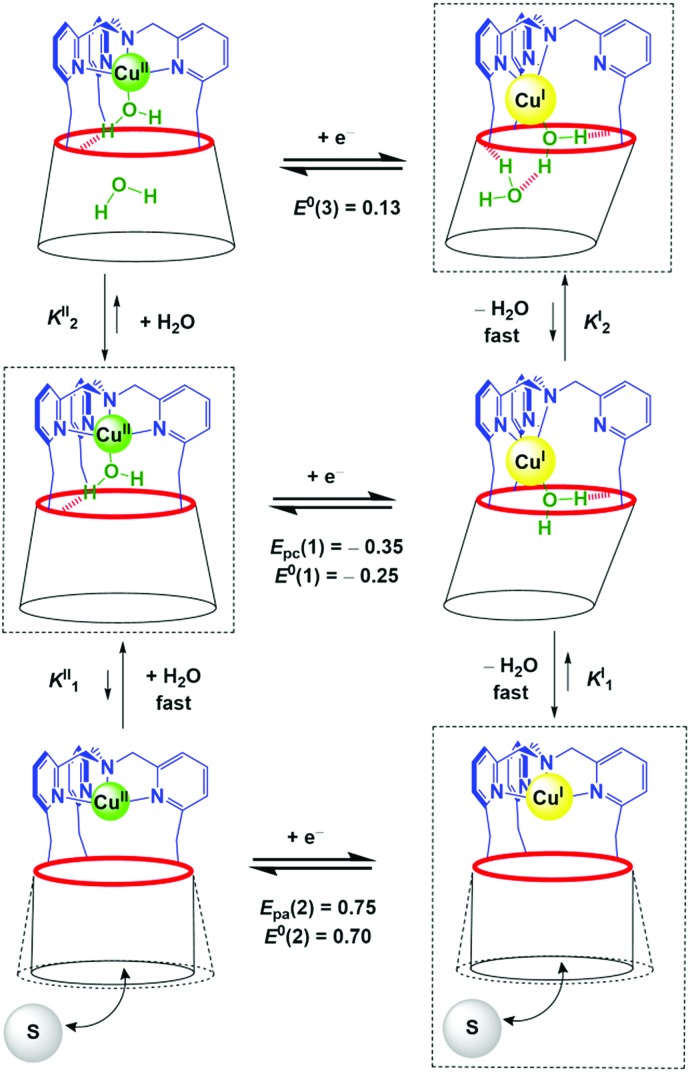
Proposed ladder mechanism of electron transfer pathways for the [Cu(calix[6]tmpa)(H_2_O)_*x*_]^*n*+^ complexes (*x* = 0, 1, 2) in CH_2_Cl_2_ under variation of the water content. The rectangles depict the thermodynamic species that have been observed by electrochemistry (*E*/*V vs.* Fc).

The detection of the *E*_pc_(2) peak only at a high scan rate (100 V s^–1^) further supports the ejection of water out of the calixarene cavity at the Cu^I^ state, leading to the water-free [Cu^I^(calix[6]tmpa)]^+^ species, and shows that water coordination at the Cu^II^ redox state is a very fast process (Fig. 4C). This redox behaviour can be visualized according to the mechanism depicted in Scheme 1 (middle and bottom parts).

### Addition of water

**Fig. 5:**
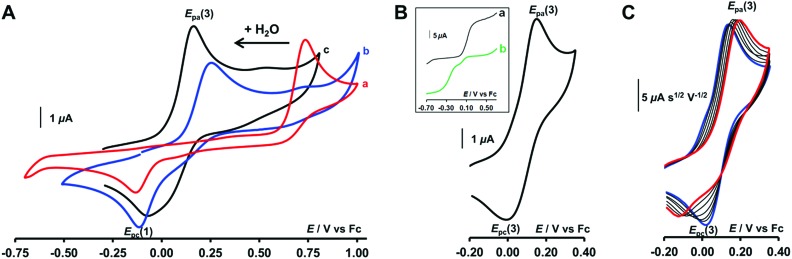
CVs at a Pt electrode in CH_2_Cl_2_/NBu_4_PF_6_ (0.1 M) at [complex] = 10^–3^ M: (A) CV at *v* = 0.1 V s^–1^ of [Cu^I^(calix[6]tmpa)]^+^ after successive addition of H_2_O: (a) 0 equiv (b), 14 equiv, and (c) 56 equiv. (B and C): CVs of [Cu^I^(calix[6]tmpa)]^+^ under H_2_O-saturated conditions (B) at *v* = 0.1 V s^–1^ and (C) for 0.02 V s^–1^ (blue) < *v* < 2 V s^–1^ (red) (*i*/*v*^1/2^ CV curves). Inset in (B): RDEV for water-saturated solutions of (a) Cu^I^ and (b) Cu^II^ complexes.

Addition of H_2_O to CH_2_Cl_2_ solutions of either Cu^II^ or Cu^I^ complexes up to saturation revealed a strong influence of the water content on the electron transfer process. As shown in Fig. 5A by CV for the Cu^I^ complex (see also Fig. S2A† for RDEV), the oxidation peak corresponding to the system at *E*_pa_(2) = +0.75 V shifted negatively with the addition of H_2_O. Upon water content increase up to saturation, it ultimately evolved to a new peak at *E*_pa_(3), corresponding to a quasi-reversible process at *E*^0^(3) = +0.13 V (Δ*E*_p_ = 135 mV at *v* = 0.1 V s^–1^; see Fig. 5B for CV and inset curve a for RDEV). The increase of the scan rate revealed a re-appearance of the reduction peak at *E*_pc_(1) at the expense of *E*_pc_(3) on the back scan (Fig. 5C). For the Cu^II^ complex, the same trend but less clear-cut was observed (ESI†). CV at various scan rates of a water-saturated solution containing the Cu^II^ complex also denoted a balance between the peaks at *E*_pc_(3) and *E*_pc_(1) for its reduction (Fig. S2B†). However *E*_pc_(1) corresponded to the stable species as confirmed by RDEV (Fig. 5B, inset curve b). After reduction down to Cu^I^, the back scan emphasized a single species by its reoxidation at *E*_pa_(3). Hence, at water saturation, the presence of a thermodynamically stable Cu^I^ redox species is observed at *E*^0^(3), and the corresponding Cu^II^ species is only transiently detected by CV (*vide infra* for kinetics). Considering that

**Scheme 2:**
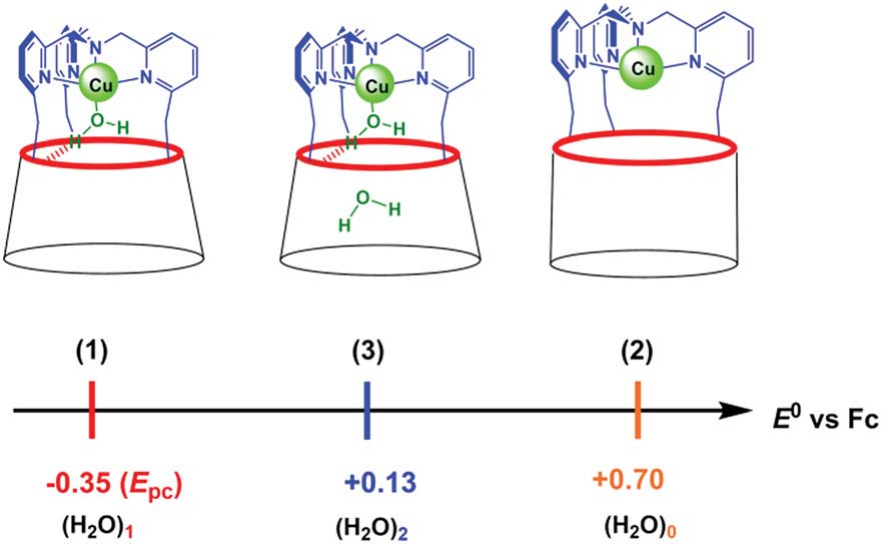
Redox potentials for [Cu^II/I^(calix[6]tmpa)(H_2_O)_*x*_]^2+/+^ in CH_2_Cl_2_/NBu_4_PF_6_. (*x* = 0, 1, 2).

(i) the redox systems (1) and (2) correspond to the mono-aqua and water-free complexes, respectively,

(ii) system (3) does not correspond to the hydroxo complex, which displays an irreversible reduction peak at a much lower potential (*E*_pc_ = - 1.34 V *vs.* Fc at 0.1 V s^–1^)^37^ (furthermore, the latter, which has been previously fully characterized by UV-vis and EPR spectroscopies in the Cu^II^ state,^37^ is obtained only after addition of a base),

(iii) system (3) is observed only at a high water content and

(iv) the hexa-*t*Bu calix[6]cone can trap and stabilize either one or two H_2_O molecules,^28^

we hypothesize that (3) corresponds to a Cu complex hosting two water molecules inside the cavity. The associated redox potentials lie in the following order: (H_2_O)_1_ < (H_2_O)_2_ < (H_2_O)_0_ (Scheme 2).

In regard to the interaction with water, the behaviour of the tmpa complexes deprived of a cavity, [Cu^II/I^(tmpa)(H_2_O)]^2+/+^, differs radically. Under dry conditions and in non-coordinating solvents, a pseudo-reversible redox process corresponding to the reduction of the 5-coordinate aqua-complex is observed at *E*^0^(1) = –0.33 V.^36^ Importantly, the associated redox system was essentially not affected by the addition of water. This indicates that the effects observed with the calixarene copper complex are not due to modifications of the medium such as the net dielectric constant. This also highlights the specific interactions between water and the calixarene cavity leading to system (3), which is not observed with the Cu-tmpa complex deprived of cavity.

### Insights into the kinetics of water exchange: ladder mechanism

Monitoring the redox process by CV at various scan rates gave further insights into the *in* and *out* mechanism for water exchange. As described above, under water-saturated conditions, the Cu^I^ state exists as a stable water-cluster adduct defined by a reversible process at *E*^0^(3). Accordingly, for a water-saturated solution of the Cu^I^ complex, only the oxidation peak of the bis-aqua species [*E*_pa_(3)] was detected at any scan rates (Fig. 5C), whereas the corresponding reversible reduction peak *E*_pc_(3) is observed at a low scan rate. For the highest scan rates, a second cathodic peak appears at the reduction potential of the Cu^II^-aqua complex [*E*_pc_(1)]. Such a behaviour characterizes a rate-limiting reaction in the Cu^II^ redox state between the two reducible species, *i.e*. a CE (Chemical–Electrochemical) mechanism:^44^ the oxidation of the Cu^I^ bis-aqua complex leads to the very fast release of the clustered guest water molecules to yield the thermodynamic Cu^II^ species, the aqua complex, that is reducible only at a low potential (*E*_pc_(1)). At a low scan rate, the timescale of the CV is long enough to allow the back-hosting of the water molecules, leading to the observation of the bis-aqua reduction peak at a higher potential [*E*_pc_(3)]. As the scan rate is increased, the back-hosting process becomes rate-limiting and the reduction of both mono-aqua and bis-aqua Cu^II^ species is observed. The situation looks a little different when starting from the Cu^II^ mono-aqua complex, which is the thermodynamic species in the +II state (Fig. S2B†). In this case, even at low scan rates, the reduction of the aqua complex detected at *E*_pc_(1) remains the major redox process observed by CV, indicating that the CE process that would lead to *E*_pc_(2) is less effective. This can result from a slow entrance step of the water molecules due to a closed conformation of the hexa-*t*Bu calix[6]cone in the Cu^II^ resting state, which slows down the mono-aqua ⇋ bis-aqua equilibrium at the Cu^II^ state. Remarkably, after the reduction step to Cu^I^, the bis-aqua cluster Cu^I^, detected at *E*_pa_(3), is observed even at high scan rates. From the electrochemical behaviour, the water-dependent redox behaviour of the cavity complexes can be rationalized by a ladder mechanism (Scheme 1). Digital voltammetric simulations based on a square scheme mechanism involving interchange between two systems allow reproducing the experimental trends and yielded the kinetic constants (see ESI†).

Assuming that *E*^0^(3) = +0.13 V, *E*^0^(1) = –0.25 V and *E*^0^(2) = +0.70 V, numerical values for *K*_*x*_^I^/*K*_*x*_^II^ can be obtained by using eqn (1) and (2), where *x* depicts the number of water molecules (*x* = 0, 1, 2):

1 *E*^0^(1) – *E*^0^(2) = *RT*/*nF* ln (*K*_1_^I^/*K*_1_^II^)

2 *E*^0^(3) – *E*^0^(1) = *RT*/*nF* ln (*K*_2_^I^/*K*_2_^II^)

These simple calculations clearly show a strong difference between *K*_1_^I^/*K*_1_^II^ (≈10^–16^) and *K*_2_^I^/*K*_2_^II^ (≈10^6^). Thus, the addition of the first water molecule to the “empty cavity” complex is much more favoured in the Cu^II^ redox state than in the Cu^I^ state. This is an expected behaviour since the Cu^II^ complex is obviously highly stabilized by catching the fifth ligand (H_2_O) in its trigonal coordination sphere, whereas Cu^I^ is already stabilized by the tmpa core when the cavity is empty. Interestingly, this ratio is reversed when more water is added: this indicates that embedment of a bis-aqua cluster stabilizes more Cu^I^*vs.* Cu^II^. At first, this seemed to be unexpected, in view of the classical behaviour of copper complexes. In fact, the presence of an additional water molecule connected to the first one through hydrogen bonding, is expected to induce an increase of the charge density on the Cu^II^ centre (which is a stronger Lewis acid than Cu^I^) and hence a negative shift of the redox system.^45^

### Mass spectrometry analysis

**Fig. 6:**
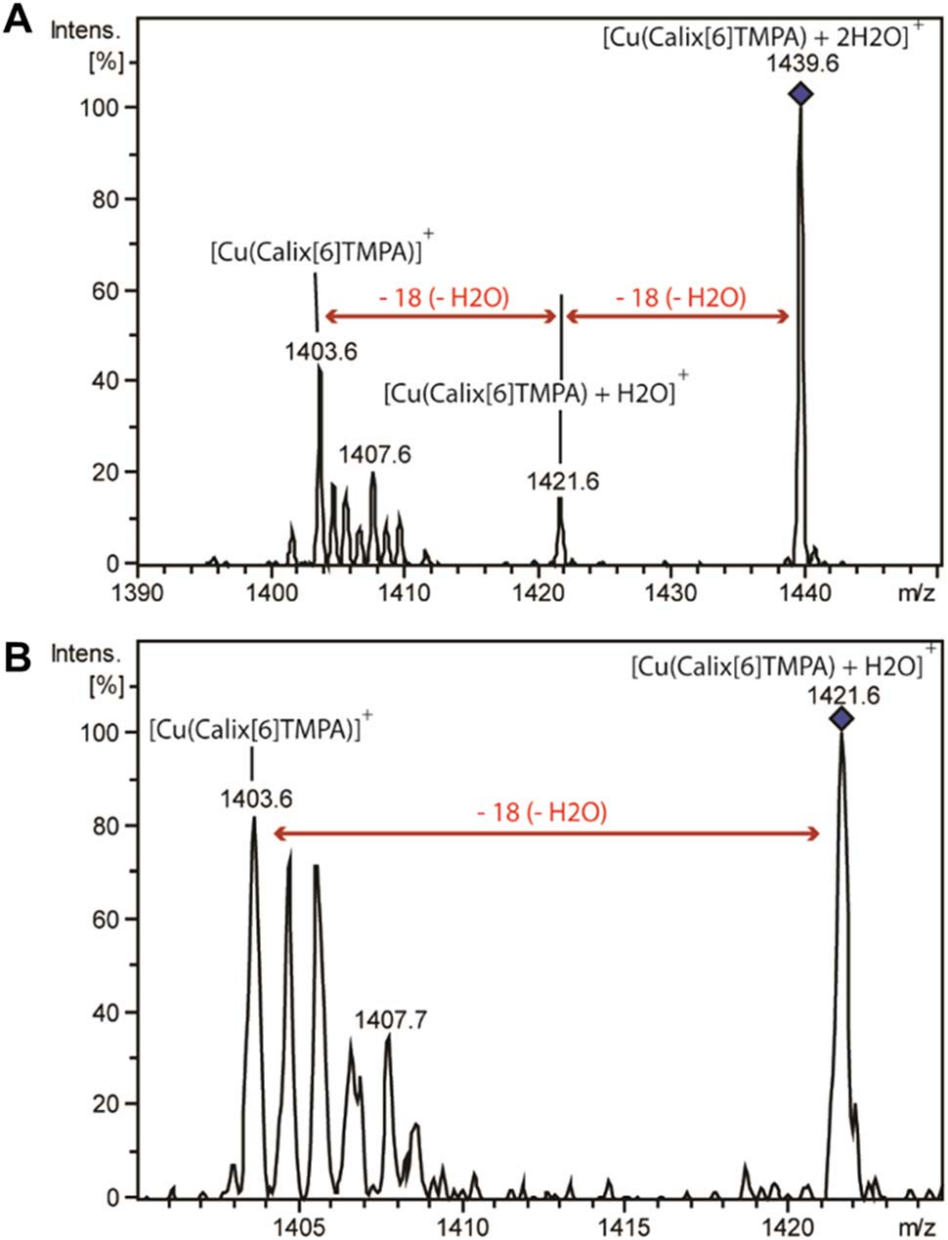
MS^2^ spectrum of [Cu(calix[6]tmpa) + 2H_2_O]^+^ at *m*/*z* 1439.6 (A) and MS3 spectrum of [Cu(calix[6]tmpa) + H_2_O]^+^ at *m*/*z* 1421.6 (B) obtained as a fragment ion of [Cu(calix[6]tmpa) + 2H_2_O]^+^ after the MS^2^ stage (*cf.* the spectrum above).

Dry and wet dichloromethane solutions of Cu^I^ complexes were mass-analysed by using an ion trap mass spectrometer. In each case, the expected empty-cavity Cu^I^ complex was observed at *m*/*z* 1403.7 with the correct relative intensities for the isotopologues (Fig. S5 and S6†). Additional clusters of peaks were detected around *m*/*z* 1422 and 1439, the relative intensity of which significantly increased with the water content (2 to 6-fold). MS^2^ experiments were then performed on the species isolated at *m*/*z* 1421.6 and *m*/*z* 1439.6 (Fig. 6).

In both cases, a neutral loss of one water molecule (–18 Da) was observed leading to the peaks at *m*/*z* 1403.7 and *m*/*z* 1421.3, respectively (along with other fragment ions from contaminants of parent ions). The fragment ion at *m*/*z* 1421.3 was further mass-selected and subjected to an additional CID stage (MS^3^ experiments, see Fig. 6): an additional 18 Da neutral loss was observed. These results confirm that the fragment ions at *m*/*z* 1421.6 and 1439.6 correspond to the mono-aqua ([Cu^I^(calix[6]tmpa)(H_2_O)]^+^) and bis-aqua ([Cu^I^(calix[6]tmpa)(H_2_O)_2_]^+^) complexes. Hence, these mass analyses demonstrate the existence of mono and bis-aqua copper complexes provided the calixarene cavity is not occupied by a bulky ligand. This further substantiates the positioning of the water ligands in the endo position, as observed for the related hexa-*t*Bu calix[6]arene-based Zn^II^ complexes displayed in Fig. 1. In order to localize the position of the bound water molecules (inside or outside the calix[6]arene cavity), the same experiment was carried out starting with the benzonitrile complex. Indeed, as shown by its X-ray structure (Fig. 3), the benzonitrile ligand fully occupies the calixarene cavity, leaving no inner space for water coordination. Mass-analysis of the complex [Cu^II^(Calix[6]TMPA)(PhCN)]^2+^ in a water-saturated dichloromethane solution showed a peak at *m*/*z* 1506.9 corresponding to the [Cu^I^(calix[6]tmpa)(PhCN)]^+^ complex with, however, no evidence of the corresponding hydrated species [Cu^I^(calix[6]tmpa)(PhCN)(H_2_O)_*x*_]^+^ (*x* = 1 or 2, see Fig. S7†). MS^2^ experiments only led to the neutral loss of PhCN at *m*/*z* 1507.9 (–103 Da).

### Insights into the structures of the aqua-Cu complexes

**Fig. 7:**
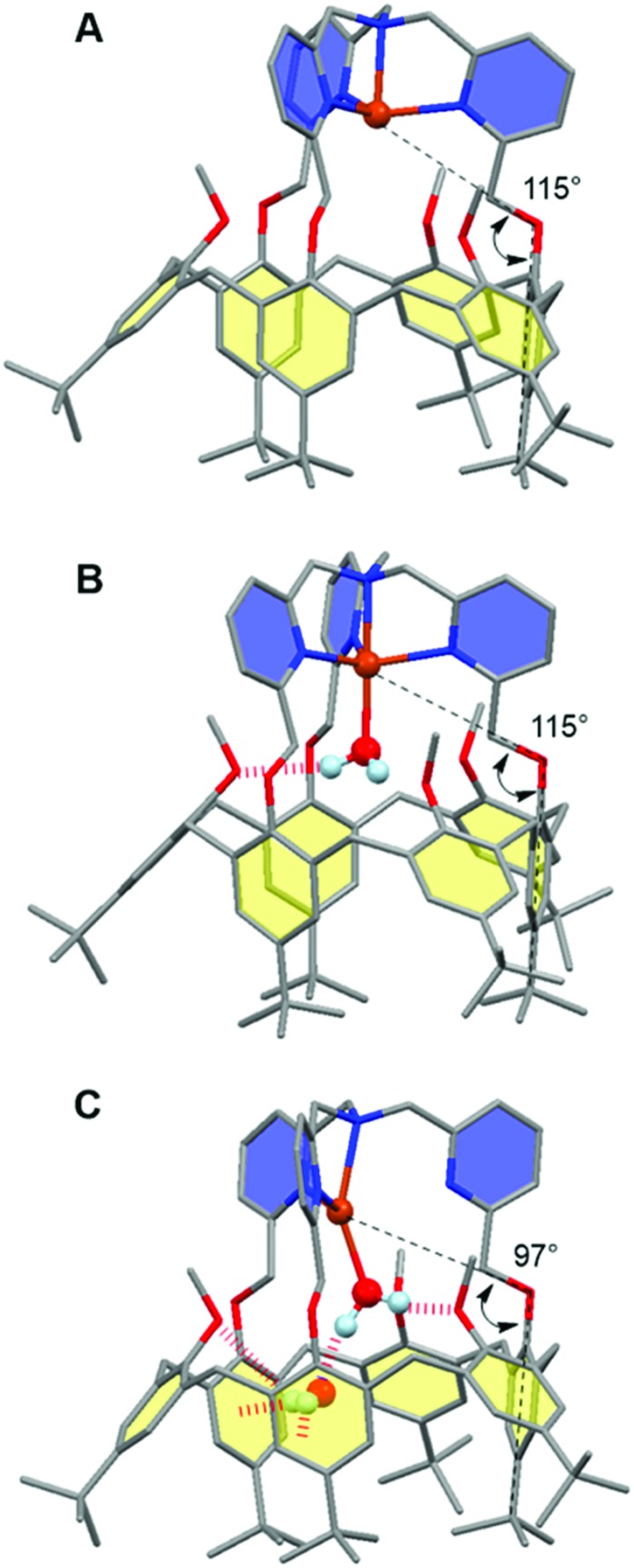
Representative snapshots extracted from the hybrid DFT/MM MDS for the [Cu^II^/^I^(calix[6]tmpa)(H_2_O)_*x*_]^2+/+^ complexes (A: Cu^I^, *x* = 0; B: Cu^II^, *x* = 1; C: Cu^I^, *x* = 2). For clarity, solvent molecules (chloroform) and hydrogen atoms are not represented except for the water molecules. The copper ion, the water molecules and the poly-aza cap were treated at the DFT level of theory, and the remaining ones were modelled using Molecular Mechanics. The black dashed lines illustrate the definition of the angles highlighting the partial inclusion of one *t*Bu group for the [Cu^I^(calix[6]tmpa)(H_2_O)_*x*_]^+^ complexes (Tables S3 and S4†). The red dotted lines indicate hydrogen and OH–π bonds. Colour code: grey = carbon, dark blue = nitrogen, red = oxygen, orange = copper, and pale blue = hydrogen.

The interplay between the inclusion of water molecules inside the cavity and the Cu^II/I^ complex stabilities was scrutinized by the Molecular Dynamics Simulations (MDS) of the [Cu^II/I^(calix[6]tmpa)(H_2_O)_*x*_]^2+/+^ complexes (*x* = 0–2) in explicit solvent (CH_2_Cl_2_). Our MDS are based on hybrid Density Functional Theory/Molecular Mechanics (DFT/MM) which enables the modifications of the coordination spheres of the Cu cations during the simulations. Details of the computational protocol can be found in the ESI.† Throughout the MDS, the water-free Cu^I^ and Cu^II^ complexes exhibited similar trigonal pyramidal geometries around the copper ion with *C*_3v_ symmetrical cavities (Fig. 7A and S17 and Table S3†). However, the introduction of one water molecule led to different behaviours for each redox state.

In the +II redox state, the water molecule binds to the copper ion in a TBP (trigonal bipyramidal) geometry, imposing a *C*_3v_ symmetry to the supramolecular system (Fig. 7B). A weak hydrogen bond between one methoxy group of the small rim and the water molecule is only occasionally formed (<50%, Table S5†). Hence, the principal source of stabilization when introducing a water molecule is the formation of the Cu^II^–OH_2_ bond.

In the +I redox state, two coordination modes involving the loose interaction of one out of the five donors (either a pyridine residue or water) are observed during the MDS with frequent interchanges (Fig. S14†). This competition suggests that a single water molecule is not particularly stabilized within the [Cu^I^(calix[6]tmpa)]^+^ complex, which is interpreted as a consequence of the well-known preference of Cu^I^ complexes for a 4-coordination mode and soft donors. Hence, the water-free Cu^I^ species is favoured over a mono-aqua adduct.

Similar features were found regarding the copper coordination sphere of the bis-aqua systems, as compared to the mono-aqua situation examined above. A TBP 5-coordination is maintained during the MDS at the +II state (Similar features were found regarding the copper coordination sphere of the bis-aqua systems, as compared to the mono-aqua situation examined above. A TBP 5-coordination is maintained during the MDS at the +II state (〈*τ*〉 = 0.89 ± 0.02, Tables S3 and S5 = 0.89 ± 0.02, Tables S3 and S5†) (ESI†). However, a 4-coordination mode was found for the +I state with the inclusion of one *t*Bu group inside the cavity and disruption of the *C*_3v_ symmetry (Table S3† and Fig. 7C). The presence of the second water molecule implies, for the +I state, (i) decoordination of a pyridine arm, (ii) strengthening of the hydrogen bond between the higher water molecule and one methoxy group compared to the mono-aqua system (Table S5†), (iii) a moderate hydrogen bond between the lower water molecule and another methoxy group, and (iv) a strong hydrogen bond between the two water molecules formed at almost 100% of the time during the MDS. The binding energy of the second water molecule amounts to –60 kJ mol^–1^ (Fig. S15†), a number that is higher in absolute values than that associated with the inclusion of the first water molecule (–42 kJ mol^–1^). Lastly, it was found that the inclusion of the third water molecule gives back a binding energy of –42 kJ mol^–1^ (Fig. S16†).

This series of values reflects the fact that a synergistic network of supramolecular interactions favours the inclusion of the second water molecule inside the cavity (Fig. 7C). In contrast, at the Cu^II^ state, the coordination of all four nitrogen atoms of the tmpa cap maintains the copper cation high in the cavity. Consequently the bound water molecule sits further away from the methoxy groups with which it only weakly interacts. In addition, the lower water molecule is not as firmly hydrogen bonded to the higher one as that in the +I state, and the second water molecule is frequently found lower in the cavity during the simulations.

In fact when DFT geometry optimizations were carried out on the [Cu^II^(calix[6]tmpa)(H_2_O)_2_]^2+^ complex, the resulting structures did not present hydrogen bonds between the water molecules (Fig. S19†). As a consequence, the energy change associated with the inclusion of the second water molecule at the +II state is almost zero. In view of the loss of symmetry suggested by molecular modelling at a high water content for the Cu^I^ state, NMR studies were conducted in CDCl_3_ with various water contents (ESI, Fig. S8–S12†). Under dry conditions, whatever the temperature is, the spectrum remained characteristic of *C*_3v_ symmetrical species. Variation of the peak widths and shifts may well be related to solvent hosting, as classically observed. Very interestingly, addition of water until saturation to the Cu^I^ complex induced a change of the spectrum towards a profile that, at low *T*, evidenced the formation of a new, non-symmetrical species for which the protons of the three pyridines are not equivalent anymore. This information is fully consistent with the hypothesized decoordination of a pyridine arm associated with the coordination of a water molecule to the Cu^I^ state. It is worth noting that decoordination of a pyridyl arm associated with the binding of an exogenous donor has been experimentally observed in the specific case of a Cu^I^-tmpa complex^46^ and that previously published theoretical calculations also predicted the decoordination of a pyridyl arm upon water binding to the Cu^I^-tmpa core.^47^ All attempts to directly or indirectly evidence a guest water molecule failed, possibly due to fast exchange with free water *vs.* the NMR time scale.^48^ Finally, the fact that this non-symmetrical species is observed only at low *T* by ^1^H NMR analysis is also consistent with an enthalpically driven water binding process as reported for the bis-aqua Zn^II^ complex.^28^

## Conclusions

Hence, on fundamental aspects, important findings emerge from the present study with regard to the modulation of the thermodynamics and kinetics of the Cu^II^/Cu^I^ electron exchange by water hosting and binding through cavity effects. The switch from an electrochemically irreversible (OFF) to a reversible (ON) state of the complex is fully controlled by the water content in the space next to the metal centre defined by the calixarene cone. Interestingly, the control of the redox potential for the bis-aqua system is essentially due to Cu^I^ stabilization. This result runs against the accepted rule, *i.e.* control by Cu^II^ stabilization.^42^ Such a supramolecular control of the redox activity by water molecules entrapped in a hydrophobic cavity actually finds echoes in biological systems. Indeed, many intermediates in biological cycles cannot be isolated, and their redox potentials are often not known, being only estimated from the resting states of the enzymes. It is shown here that the insulating effect of the cavity can yield transiently very strong oxidants and reductants under dry conditions. Hence, the specific design of the coordination spheres within a cavity surrounding the copper ion allows the control of the redox properties of the system by simple variation of the water content.

## Conflicts of interest

There are no conflicts to declare.

## Supplementary Material

Supplementary information

Crystal structure data
