# Supplementary material for: Gating the electron transfer at a monocopper centre through the supramolecular coordination of water molecules within a protein chamber mimic
Source: Chem Sci. 2018 Aug 30;9(43):8282–90. doi: 10.1039/c8sc03124j (PMC6240898; doi:10.1039/c8sc03124j)
Supplement: Supplementary file 1 [file SC-009-C8SC03124J-s001.pdf]

Electronic Supplementary Material (ESI) for Chemical Science.
This journal is © The Royal Society of Chemistry 2018

Supporting Information

for

Gating the electron transfer at a monocopper centre through the supramolecular coordination of water molecules within a protein chamber mimic

Nicolas Le Poul,[a] Benoit Colasson,[b] Grégory Thiabaud,[b] Dany Jeanne Dit Fouque,[a] Claudio Iacobucci,[a] Antony Memboeuf,[a] Bénédicte Douziech,[a] Jan Řezáč,[c] Thierry Prangé,[d] Aurélien de la Lande,[e] Olivia Reinaud*[b] and Yves Le Mest*[a]

[a] *Laboratoire de Chimie, Electrochimie Moléculaires et Chimie Analytique, UMR CNRS 6521, Université de Brest, 29238 Brest, France.*

[b] *Laboratoire de Chimie et Biochimie Pharmacologiques et Toxicologiques, UMR CNRS 8601, Université Paris Descartes, 75006 Paris, France.*

[c] *Institute of Organic Chemistry and Biochemistry, Academy of Sciences of the Czech Republic, Flemingovonám. 2, 166 10 Prague 6, Czech Republic.*

[d] *Laboratoire de Cristallographie et de Résonance Magnétique Nucléaire. Biologiques (CNRS UMR 8015), Université Paris Descartes, 4, Avenue de l'Observatoire, 75006 Paris, France.*

[e] *Laboratoire de Chimie Physique, UMR CNRS 8000, Université Paris Sud, 91405 Orsay, France.*

Table of contents

Materials and methods **S2**

Tables, schemes and figures.. **S7**

References.. **S21**

1. Materials and Methods

Electrochemical and spectroscopic studies. The electrochemical studies of the copper complexes have been performed in a glovebox (Jacomex) (O_2 < 1 ppm, H_2O < 1 ppm) with a home-designed 3-electrodes cell (WE: Pt, RE: Pt in a Fc^+/Fc equimolar solution, CE: Pt). The potential of the cell was controlled by an AUTOLAB PGSTAT 100 (Ecochemie) potentiostat monitored by a computer. Data acquisition was performed with NOVA software. “Extra-dry” dichloromethane (H_2O < 50 ppm, Acros) was used as received and kept under N_2 in the glovebox. $CDCl_3$ was purchased from Euriso-top and contains less than 100 ppm of water. The supporting salt, NBu_4PF_6, was purified, dried under vacuum for 48 hours at 100° C, then kept under N_2 in the glovebox. All experiments have been performed with solutions of *c.a.* 0.001 mol L^{-1} in electroactive species. ^{1}H NMR spectra were recorded on an Advance 500 spectrometer (500 MHz).

Synthesis. Calix[6]tmpa ligand, $[Cu^{II}(tmpa)(H_2O)](CF_3SO_3)_2$, $[Cu^{II}(calix[6]tmpa)(H_2O)](ClO_4)_2$ and $[Cu^{I}(calix[6]tmpa)](OTf)$ complex were prepared as previously reported.[1,2,3]

Voltammetric simulation. This section provides information about the procedure and estimations of the parameters. Numerical simulations of the voltammograms were performed with the BAS DigiSim© simulator 3.03 (Bioanalytical Systems), using the default numerical options with the assumption of planar diffusion (*D* assumed as 10^{-5} cm^2 s^{-1} for all species) and a Butler-Volmer law for the electron transfer. The charge-transfer coefficient (α) and the electron transfer standard rate constant (k^0) were taken as 0.5 and 10^4 cm s^{-1} respectively. On the basis of the ladder mechanism depicted in Scheme S1, the thermodynamic and kinetic parameters for the chemical reaction involving the water exchange reaction have been gathered in Tables S1 and S2. They were determined from the good matching between the simulated and experimental voltammograms for different scan rates and H_2O concentrations (dry (1 mM) and wet-saturated (100 mM)). The ladder mechanism is based on the experimental observation of three redox systems at $E^0(1)$= -0.25 V, $E^0(2)$= 0.70 V and $E^0(3)$= -0.13 V.[4] On this assumption, the general trends observed experimentally were reproduced by voltammetric simulation at both Cu^I and Cu^{II} redox states. Indeed, in dry conditions (1 mM), the irreversible anodic and cathodic peaks were obtained from the simulation program for the exchange of one molecule of water between the mono-aqua and water-free cavity complexes (Figure S3A and S3C). The thermodynamic constants related to this process, K_1^I and K_1^{II}, display the high affinity of Cu^{II} and low affinity of Cu^I for H_2O. The kinetics constants show the fast ejection of water at Cu^I (k_{b1}^I) and re-coordination of water at Cu^{II} ((k_{f1}^{II}). This ejection effect at Cu^I is inherent to the calix[6]arene cavity since the monoaqua system is quasi-reversible for the Cu-tmpa analogous complex. At high scan rate (100 V s^{-1}), reversibility can be obtained for the water free system (Figure S4), whereas the mono-aqua system remains irreversible. When the water content was increased until saturation (100 mM), a new quasi-reversible was observed at $E^0(3)$ = +0.13 V. This new system was ascribed to the bis-aqua adduct. Simulated CVs were obtained by using thermodynamic constants such that $K_2^I >> K_2^{II}$. The high value obtained for K_2^I shows the high stability of the Cu^I bis-aqua complex. At Cu^{II}, the K_2^{II} value (close to unity) displays the relative equal stability of the bis-aqua vs mono-aqua species. Kinetically, an interesting parameter is k_{f2}^I which gives the high rate of the associative process for the formation of the bis-aqua Cu^I complex from the mono-aqua.

Mass spectrometry. A stock solution of $[Cu^{I}(calix[6]tmpa)]^{+}$ complex (0.01 mM) was prepared in dry degassed HPLC grade dichloromethane in the glovebox (H_2O < 1ppM, O_2 < 1ppm). Half of this stock solution was used as prepared (solution (1)), whereas the other half was saturated with degassed deionized ultrapure water (18.2 MΩ cm) (solution (2)). Additionally, a stock solution of $[Cu^{I}(Calix[6]tmpa)]^{+}$ complex (1 mM) was prepared from dry and degassed benzonitrile in the glovebox. This solution was first diluted with dry dichloromethane (0.01 mM) and saturated with deionized ultrapure water (solution (3)).All samples were prepared in glovebox under argon and injected in the mass spectrometer without having being in contact with air.
Mass spectrometric experiments were carried out using an ion trap mass spectrometer (HCTplus, Bruker Daltonics) equipped with an electrospray ionization source (ESI, Agilent Technologies) and operated in the positive ion mode. The solutions were introduced by direct injection, using a syringe pump, to be electrosprayed at a 50 µL/min flow rate. N_2 was used as nebulizing (P=10 psi) and drying gas (5 L/min) heated at 150°C. Helium was used as the trapping and collision gas at a 1.40 10^{-5} mbar pressure (uncorrected gauge reading). MS^n spectra of the complex with one ($[Cu(calix[6]tmpa) + H_2O]^{+}$ at m/z 1421.7) and two ($[Cu(calix[6]tmpa) + 2H_2O]^{+}$ at m/z 1439.7) water molecules were performed with the following main parameters: capillary voltage 3.5 kV, end plate -0.5 kV, skimmer 40 V and a trap drive at 120. MS analysis was followed by a 200 ms isolation time to isolate the complex with one/two water molecules using 1.5/1.0 Th isolation windows respectively. Collisional excitation was then performed during 100 ms using a 10 Th excitation width. Data acquisition and mass spectra processing were done using DataAnalysis 3.3 software (Bruker Daltonics, Bremen, Germany).

NMR study on the influence of D_2O concentration. We have then studied the influence of the water concentration on the structure of the complex in order to characterize the nature of the interactions between the complex and water. The water concentration was increased by adding to the initial $CDCl_3$ solution a water-saturated $CDCl_3$ solution. ^1H NMR spectra were recorded after each addition at 4 different temperatures (263 K, 273 K, 300 K and 320 K). As soon as a small amount of water was added into the NMR tube, the spectra changed quite impressively at low *T*. Figure S8 displays the four spectra recorded at the four temperatures after the addition of 140 µL of water-saturated $CDCl_3$. A careful comparison of the spectra obtained before and after the addition of D_2O at high and low *T* shows that, while no change is observed at 320 K, new peaks denoting a new species have grown at 263 K after addition of D_2O (Figures S9, S10, S11 and S12). These changes are not the result of dilution as adding "normal" $CDCl_3$ did not modify the NMR profiles.
Hence, when water is added, a new species appears and progressively grows as the *T* is decreased. These news signals are observed on the whole window of the spectrum but are particularly distinguishable for the resonances corresponding to the protons of the pyridyl units (around 8 ppm, Figure S12), the $ArCH_2$ bringing the aromatic units of the calixarene (around 4 ppm, Figure S8) and the *t*Bu groups (around 1 ppm, Figure S10). All together they attest to the formation of a non-symmetrical complex with partial inclusion of two *t*Bu groups as more specifically indicated by the peaks at 0.47 and 0.66 ppm.
All these observations are consistent with the formation of a species presenting a coordination bond to a small guest (here water) and the decoordination of one pyridyl arm which in turn allows the partial inclusion of one *t*Bu (see Scheme S2 and the Molecular Dynamic Simulations section below).

Hybrid DFT/MM Molecular Dynamics Simulations. The thermally accessible molecular configurations for the complexes $[Cu^{I}(calix[6]tmpa)(H_2O)_x]^{2+/+}$ (x = 0, 1, 2) have been sampled by means of hybrid DFT/MM (Density Functional Theory/Molecular Mechanics) molecular dynamics simulations. Each complex was solvated in a cubic box of chloroform molecules of 55 Å edge. The QM partition was comprised of the copper cation, the TMPA moiety and the water molecules (when present). The remaining calixarene atoms, the counter-ions and the solvent molecules were treated at the Molecular Mechanics level. Our QM/MM approach follows a subtractive scheme[5] with an electrical embedding technique, *i.e*. the DFT computations are realized in the presence of the electric field created by were environment. DFT calculations were performed with demon2k[6] while MM calculations were performed with the software CHARMM.[7] We have included atom charges situated at distances lesser than 10 Å from the QM part. The QM/MM boundaries have been treated with link atoms, the bond lengths of which were previously optimized at the full DFT level. The Perdew Becke Ernzerhof (PBE)[8] functional has been used for the DFT part in conjunction with the all electron basis set DZVP-GGA (Double Zeta Valence plus Polarization functions for Generalized Gradient Approximation) and the GEN-A2 auxiliary basis set.[9] An empirical dispersion correction to the DFT energies has been added for the O, N, C and H atoms.[10,11] We performed 1ns of classical MD simulations to relax the system before starting the hybrid DFT/MM simulations. The latter are carried out within the canonical ensemble, the temperature of which (300 K) is controlled using a Nose-Hoover thermostat with a friction coefficient of 0.1ps. The time step is set to 0.5 fs. The atoms located beyond 15 Å from the calixarene were frozen during the MDS. For each simulation the system was relaxed during 1ps before accumulating data for analysis. We note that all structures were close to C3v symmetry when starting the hybrid DFT/MD MDS. Between ca 7 ps and 11ps of simulation have been gathered to investigate the dynamics of the copper coordination at different redox states and with different number of water molecules. The differences in simulation lengths between each system were due to computational limitations.
The statistical analysis of the MD simulations outputs were realized with "*The R Project for Statistical Computing*" and the "*coda*" package.[12,13] The expectation values for the coordination characteristics at the copper center (Table S3-S5) have been estimated from their average values and standard deviations on the ensemble of snapshots extracted from the hybrid DFT/MD simulations. The 95% confidence interval is then obtained using the formula (in the case of a variable ε):

$$\langle \varepsilon \rangle = \bar{\varepsilon} \pm 2\frac{\sigma}{\sqrt{n\rho}}$$

where $\bar{\varepsilon}$ is the average value of ε taken over *n* configurations extracted from the MDS, σ is the standard deviation and ρ is the chain statistical efficiency.[14] The value of ρ can be estimated from the analysis of the autocorrelation function of ε using, for example, the aforementioned R-coda package.

Computational studies. Binding energies of water within the calixarene cavity. To estimate the binding energies of the inclusion of water molecules within the cavities we have performed various geometry optimizations of the complexes in gas phase at the full DFT level. All these computations were used with deMon2k. Various optimizations with distinct starting structures were performed in agreement with the variety of structures encountered in the MDS. For each

optimized system the binding energies of the water molecules within the cavity ΔE_{bind} are computed according to

$$\Delta E_{bind} = E[Cu^{II}(calix[6]tmpa)(H_2O)_n]_{opt} - E[Cu^{II}(calix[6]tmpa)]_{opt} - n.E[H_2O]_{opt}$$

where $E[X]_{opt}$ is the energy of molecule X in its optimized geometry. For all computations we used the all electrons DZVP-GGA basis set and the GEN-A2 auxiliary basis set coupled to the PBE functional. A multipole expansion technique was employed to compute the long range integrals of the coulomb operator. A grid of FINE accuracy was used.

X-Ray structure of the $[Cu^{II}(calix[6]tmpa)]^{2+}$ benzonitrile complex. Crystals suitable for X-ray diffraction analysis were obtained by slow diffusion of Et_2O vapor into a $CHCl_3$ solution. Crystals were not stable upon standing to air, they were rapidly transferred in paraffin oil and fished out with a Hampton® cryoloop then rapidly quenched in liquid nitrogen. All data recordings were performed at ESRF synchrotron in Grenoble, France, beam line BM30. The wavelength was fixed to 0.964 Å, and the resolution set at the maximum available by the geometry of the detector in use (Quantum ADSC 315r). The crystals are triclinic, space group P-1 with parameters: a=30.097(1) Å; b=30.866(1) Å; c=31.958(1) Å; α=91.51(4); β=102.51(4)°; γ=94.11(4); There are 4 independent molecules in the asymmetric unit, accounting for more than 400 atoms. Because of the low symmetry and the method used in data recordings (single ω rotation), two crystals with different orientations were used. In each case, a full rotation range of 360° was adopted. For the two crystals, 455277 reflections were recorded and processed, then scaled and reduced to 58216 independent reflections, using MOSFLM program[15] interfaced with the CCP4 suite of programs.[16] These data in the range 15-0.98 Å were used for structure determination and refinements.

Structure determination was performed by direct methods with the SHELXD program[17] using a sub-structure taken from the already solved calix[6]tmpa.[18] The best solution was submitted to the automated E-map refinement as coded in the DIRDIF program.[19] This resulted in the complete development of the four independent molecules except for some of the ter-butyl groups found highly agitated, even at 100 K. These ter-butyls were further idealized and refined as rigid groups. Refinements were conducted with the SHELXH program[17] using isotropic individual thermal parameters, then anisotropic. Hydrogens were imposed at their theoretical places but not refined. Refinements converged to R=11.23 % for 45966 observed *F*'s (with $F \geq 4\sigma(F)$) and R=12.1 % for all 58216 *F*'s.

The final structure consist of four molecules of Cu^{II}calix[6]tmpa-benzonitrile complexes plus 8 perchlorate counter ions and three solvated benzonitrile molecules in the packing together with an additional molecule of disordered chloroform. Four residual peaks were modeled as two additional methanol molecules. The four Cu-calix[6]tmpa molecules have similar cone conformation but the fourth display the half-cone conformation with one aromatic ring of the calix[6] reverted.

The structural features are close to those obtained for the CH_3CN analogous Cu^{II} complex $[Cu^{II}(calix[6]tmpa)(CH_3CN)]^{2+}$. The solid state analysis of the benzonitrile complex shows a strong axial (τ = 0.98)[20] trigonal bipyramidal (TBP) Cu^{II} center bound to the tmpa cap and to a benzonitrile molecule. The nitrilo part of the guest aligned on the C_3 axis in the heart of the calixarene cavity. The benzyl moiety is slightly tilted due probably to π–π interactions with one anisole of the calixarene cavity. The comparison of the two complexes shows that the calixarene core is slightly affected by the guest exchange. Indeed, the average *t*Bu-*t*Bu distances

(taken between two quaternary carbon atoms of the tBu groups attached to aromatic units linked to the cap) are 6.22 Å and 7.68 Å for the CH_3CN and PhCN adducts, respectively. Similarly, the average O-O distances between the methoxy groups are 6.01 Å (CH_3CN) and 6.19 Å (PhCN). The flexibility of the cavity and its adaptability towards a more or less large guest can also be quantified with the calculation of the inner cavity volume which increases by 11% when CH_3CN is replaced by PhCN (*vide infra*).

Determination of the volume of the cavities. To evaluate the plasticity of the calix[6] system, the cavities built by the t-butyl phenyl rings in the cone conformation were measured in the three available tmpa structures, the apo form (empty), the Cu-acetonitrile complex, and the Cu-benzonitrile complex, respectively. Several programs are available for that purpose,[21,22,23,24] they all of them rely on an equivalent algorithm: the rolling of a sphere, (usually a water molecule with a fixed Van der Waals radius (1.4 Å) on the surface determined by all the atoms lining the interior of the cavity. They give somewhat different results as the method is very sensitive to the parameters in use (Van der Waals radii, etc ..). Whatever the program in use, one can obtain reliable values as long as one stay on the same protocol in the calculations for the three structures in comparison.

The program we used was voidoo[21], a program more dedicated to protein structures but easy to extend to other kind of molecules, by tailoring its library to cope with the tmpa molecular structure. For strict comparisons, the three files, apo, acetonitrile, and benzonitrile complexes were first prepared following the same protocol: the apo structure (empty calix[6]tmpa) has an additional cavity delimited by the three pyridines on top which increases the real size of the cavity delimited by the lower calix[6] arene moiety; hence a copper ion was artificially added to restrain the cavity to what it should correspond in the two others. In addition, as the cavities are nearly open to the solvent at the level of the terbutyl groups, an additional « ghost atom » was set as a closure at the level of the terbutyl groups and located on the three-fold axis of the tmpa as to allow reliable calculations in the three structures (Figure S20). Indeed, the complex structures were considered void of their acetonitrile or benzonitrile ligands in all the calculations.

The results are reported in the Table S6. It is important to note that in the case of the apo structure, three copies of the calix[6]tmpa molecule are present in the asymmetric unit, while only one is present in the acetonitrile complex, and four are independently observed in the benzonitrile structure. All of them are in cone conformation except in the case of the third copy of the apo form. The last column of the Table S6 reports the averaged value obtained when several copies are present, the error is evaluated as two to three times the esd's on individual values.

-Calix[6]tmpa apo structure: As mentioned previously, the third molecule has a complete different conformation with two of the tBu benzene rings reverted. The cone conformation is no longer respected and no cavity can be found in that case.

-Cu-calix[6]tmpa benzonitrile complex: In the third independent copy, the hosted benzonitrile shows clearly two orientations in the cavity, nearly perpendicular to each other, both aligned along the cone axis. As a consequence, the cavity is enlarged compared to the three other copies. The result in that case was not included in the average.

2. Tables, schemes and figures

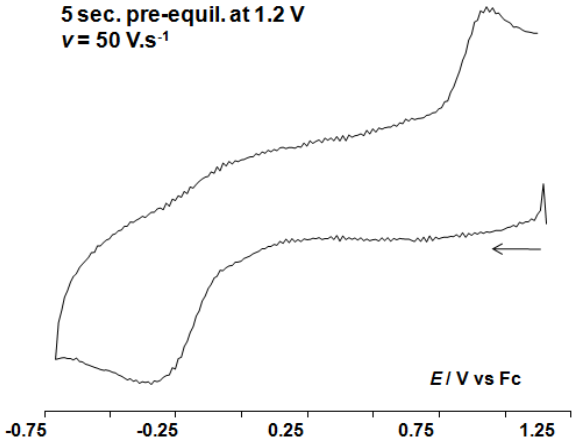

Figure S1. Cyclic voltammogram at a Pt electrode in anhydrous CH_2Cl_2/NBu_4PF_6 0.1 M + 10^{-3} M $[Cu^{II}(calix[6]tmpa)(H_2O)]^{2+}$ at v = 50 V s^{-1} for 1 cycle starting from 1.20 V with 5 seconds pre-equilibrium at this potential.

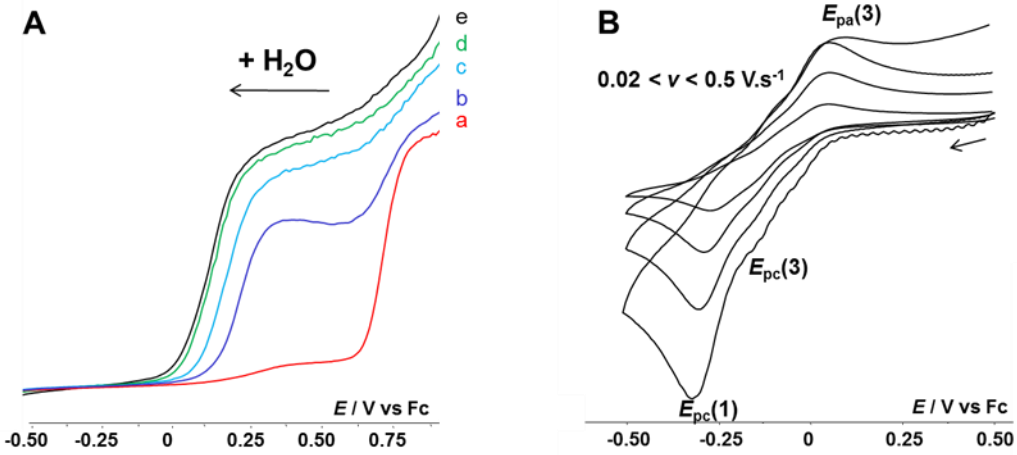

Figure S2. A) RDEV at a Pt electrode in dry CH_2Cl_2/NBu_4PF_6 solution of $[Cu^{I}(calix[6]tmpa)]^{+}$: From a) to e) progressive addition of H_2O up to saturation; B) Cyclic voltammetry (0.02 V s^{-1} < v < 1 V s^{-1}) a Pt electrode in a fresh H_2O-saturated CH_2Cl_2/NBu_4PF_6 solution of $[Cu^{II}(calix[6]tmpa)(H_2O)]^{2+}$.

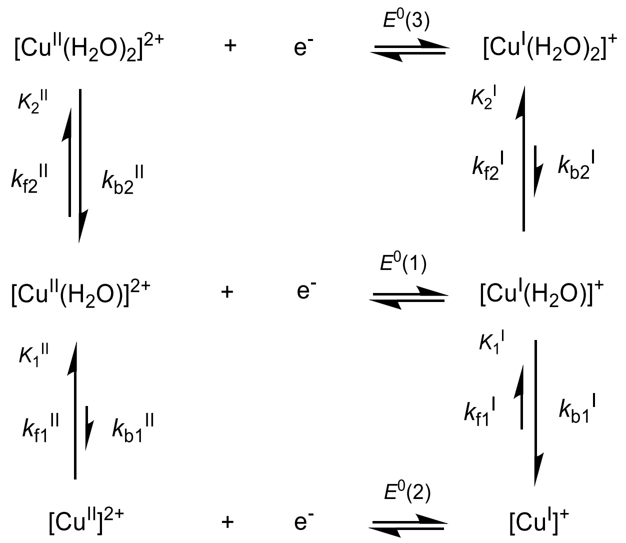

Scheme S1. Ladder mechanism for H_2O exchange at the Cu^{II}/Cu^{I} redox process for the $[Cu^{II/I}(calix[6]tmpa)(L)]^{n+}$ complexes (L=.CH_2Cl_2, H_2O, $(H_2O)_2$ where "c" stands for cluster).

Table S1. Thermodynamic parameters (equilibrium constants and redox potentials) from the simulation of experimental voltammograms (0.1 V s^{-1}) for $[Cu^{II/I}(calix[6]tmpa)(L)]^{n+}$ complexes ((L = CH_2Cl_2, H_2O, $(H_2O)_2$) according to scheme S1. *E* /V *vs* Fc.

**Parameter**	**E^0(1)**	**E^0(2)**	**E^0(3)**	**K_1^{I}**	**K_2^{I}**	**K_1^{II}**	**K_2^{II}**
Value	-0.25	+0.70	+0.13	$4\ 10^{-4}$	$1.9\ 10^{6}$	$5\ 10^{12}$	0.7

Table S2. Kinetic parameters from the simulation of experimental voltammograms for $[Cu^{II/I}(calix[6]tmpa)(L)]^{n+}$ complexes (L=.CH_2Cl_2, H_2O, $(H_2O)_2$) according to scheme S1. *k* /M^{-1} s^{-1}

**Parameter**	**k_{f1}^{I}**	**k_{b1}^{I}**	**k_{f2}^{I}**	**k_{b2}^{I}**	**k_{f1}^{II}**	**k_{b1}^{II}**	**k_{f2}^{II}**	**k_{b2}^{II}**
Value	$5.0\ 10^{2}$	$1.1\ 10^{6}$	$2.0\ 10^{10}$	$1.1\ 10^{4}$	$1\ 10^{5}$	$2\ 10^{-8}$	$1.0\ 10^{2}$	$1.4\ 10^{2}$

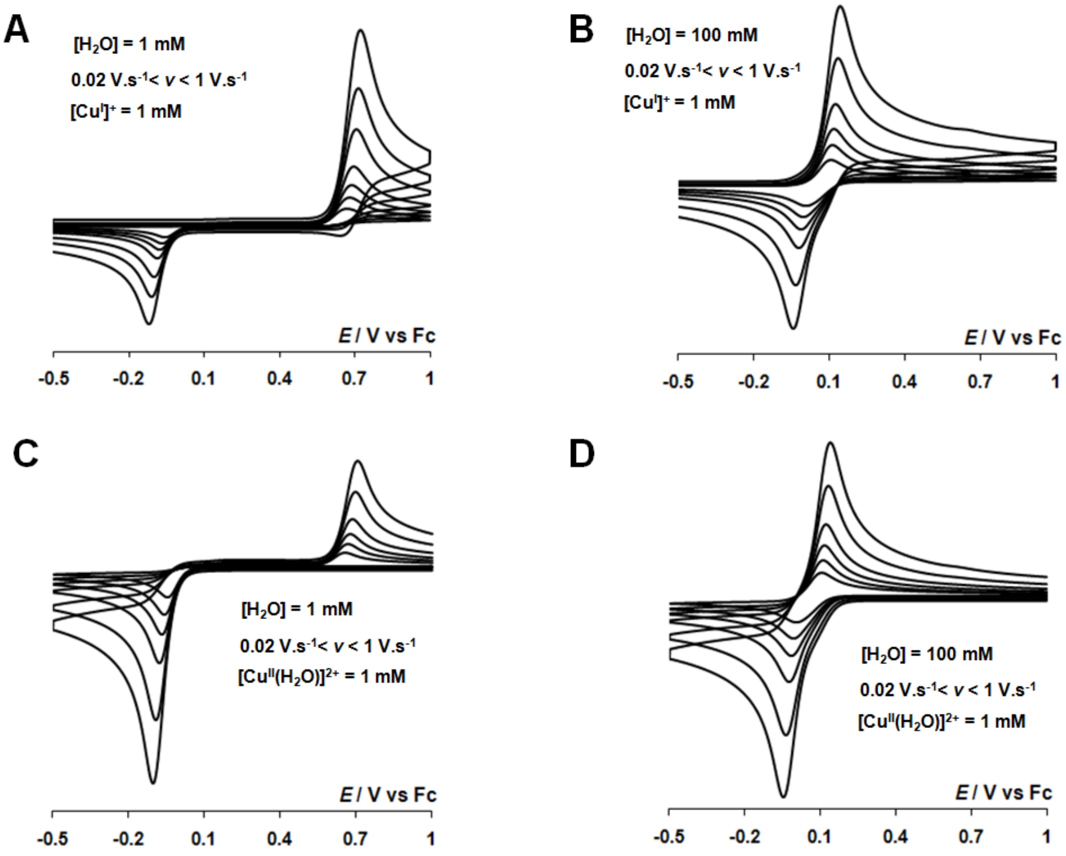

Figure S3. Simulated cyclic voltammograms (0.02 V.s^{-1} < v < 1 V.s^{-1}) on the basis of the ladder mechanism proposed in scheme S1 for A) and B) [Cu^{I}(calixtmpa)(L)]$^{+}$ and C) and D) [Cu^{II}(calixtmpa)(L)]$^{2+}$ complexes under dry ([H_2O]=1 mM) and wet ([H_2O]=100 mM) conditions (R_{unc} = 100 Ω, C_{dl} = 1.5 µF).

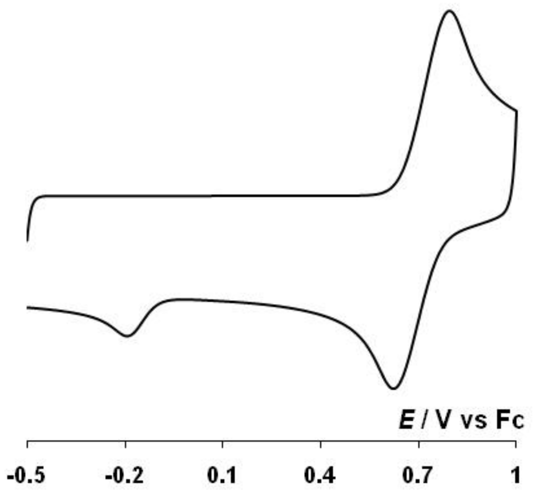

Figure S4. Simulated cyclic voltammogram (v = 100 V.s^{-1}) on the basis of the ladder mechanism proposed in scheme S1 for [Cu^{I}(calixtmpa)(L)]$^{+}$ under dry ([H_2O]=1 mM) conditions (R_{unc} = 100 Ω, C_{dl} = 1.5 µF).

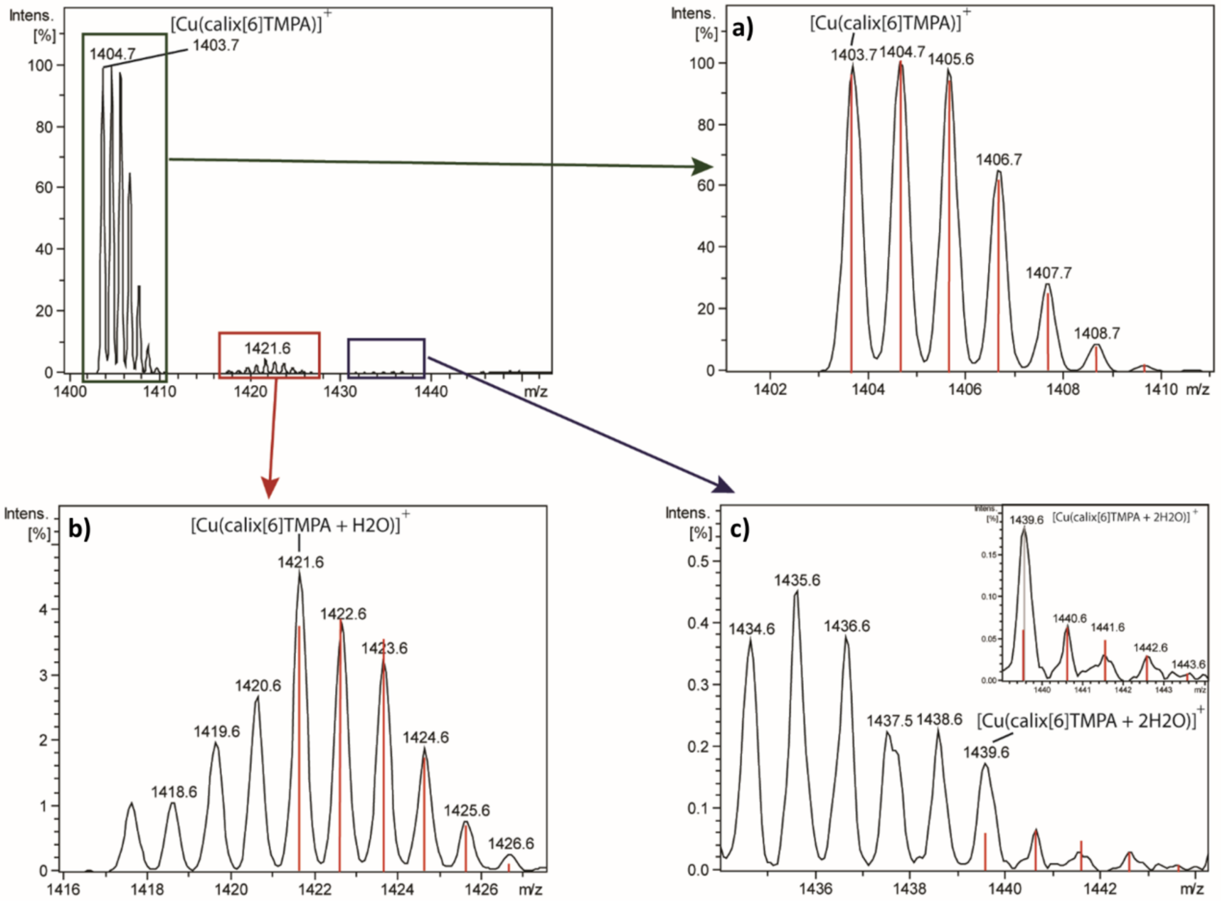

Figure S5. MS spectrum of the monocopper complex [Cu(calix[6]TMPA)]$^+$ in dry CH_2Cl_2 with straight red lines as theoretical profiles.

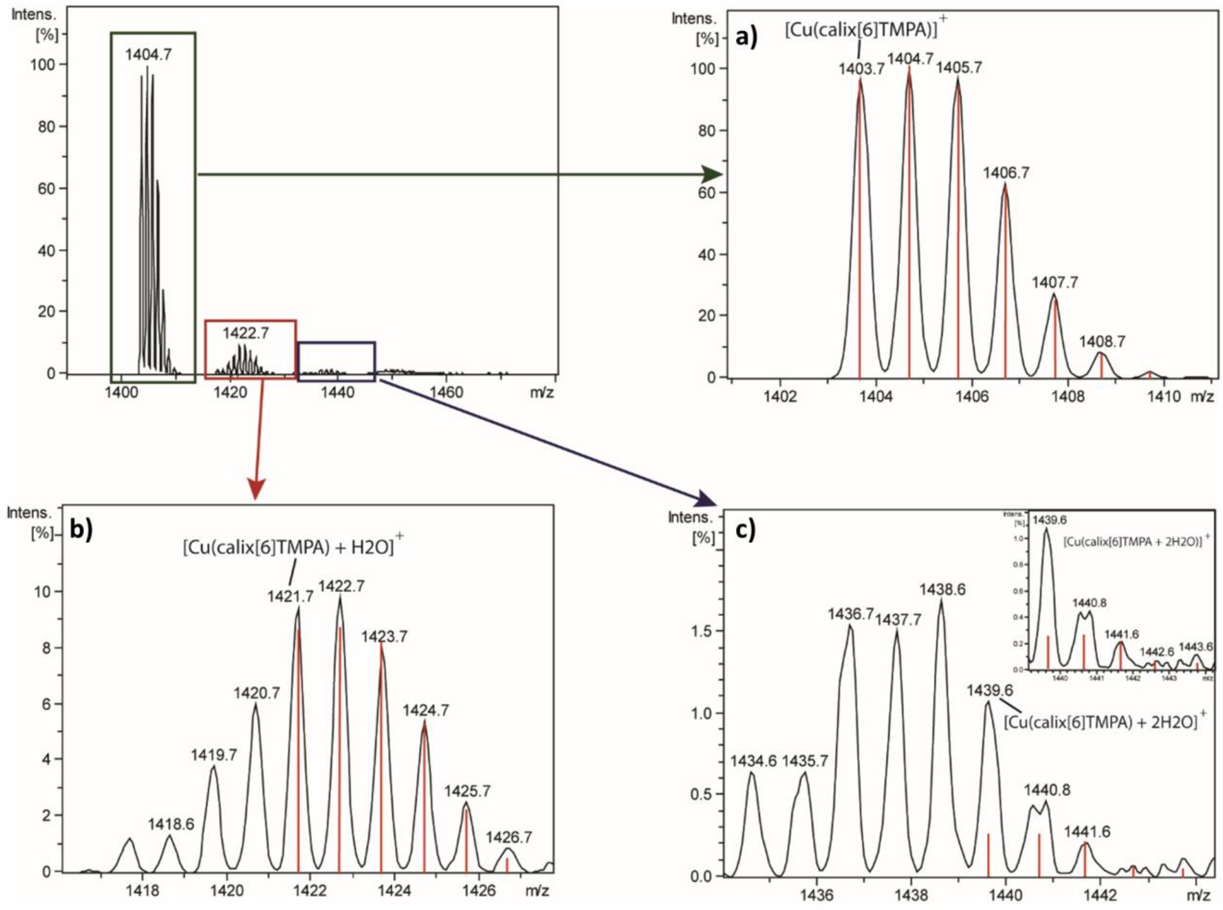

Figure S6. MS spectrum of the monocopper complex [Cu(calix[6]TMPA)]$^+$ in wet CH_2Cl_2 with straight red lines as theoretical profiles.

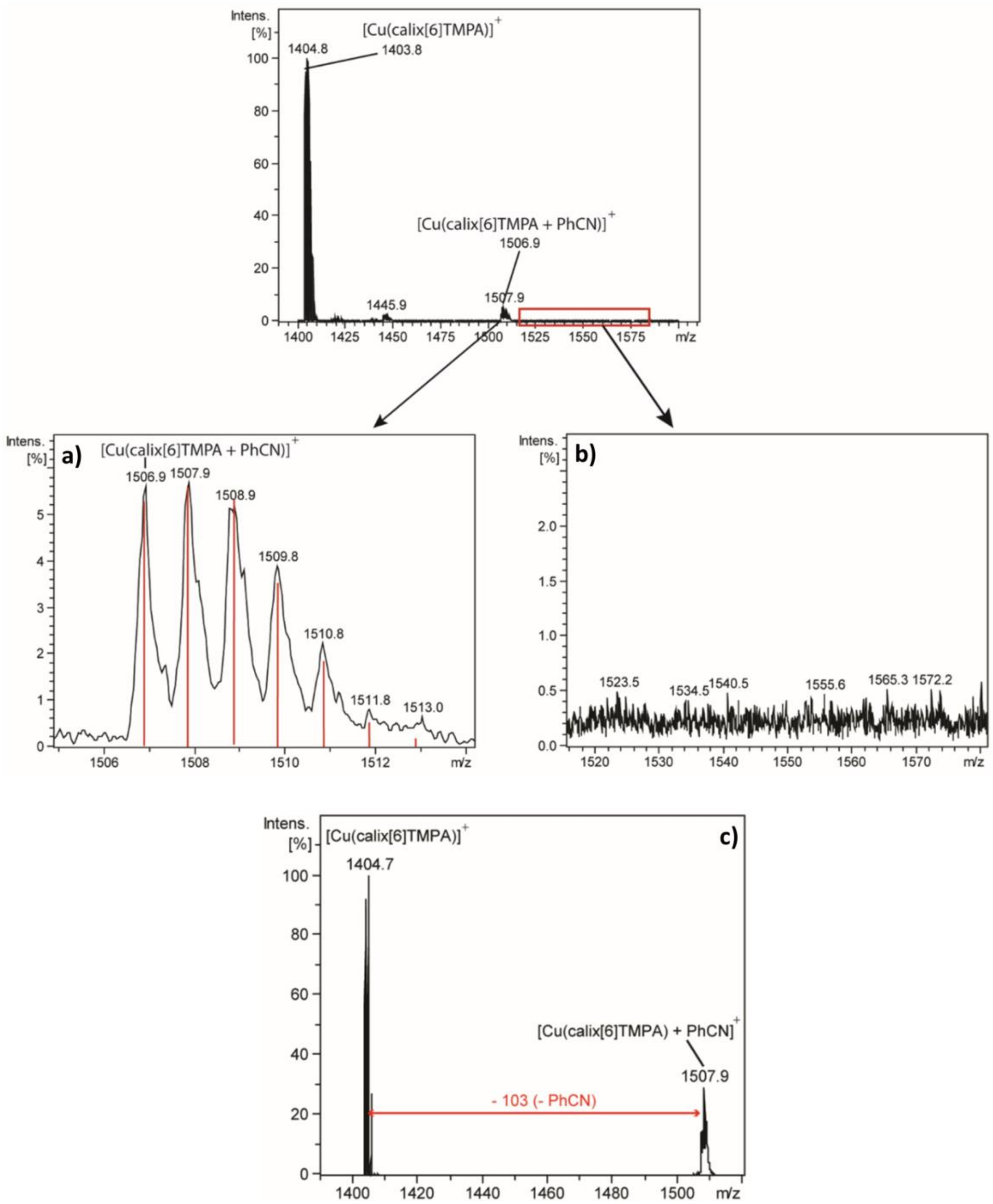

Figure S7. MS spectrum of the monocopper complex [Cu(calix[6]TMPA)]$^+$ in wet dichloromethane with PhCN with straight red lines as theoretical profiles (Fig. a). No coordination of additional water molecule is observed along with PhCN (Fig. b). MS^2 spectrum of [Cu(Calix[6]TMPA) + PhCN]$^+$ at m/z 1507.9 (Fig. c).

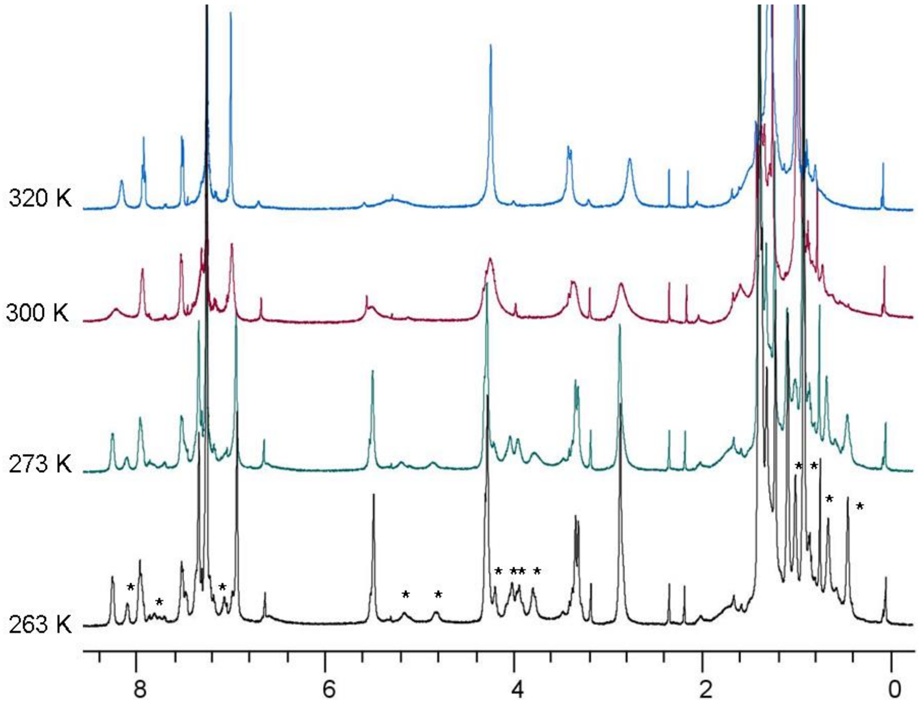

Figure S8. ^1H NMR of $[Cu^I(calix[6]tmpa)](OTf)$ ($CDCl_3$; 500MHz) after addition of 140 µL of D_2O-saturated $CDCl_3$ at 263 K, 273 K, 300 K and 320 K (the resonances of the growing non-C_{3v} symmetrical species are indicated with *).

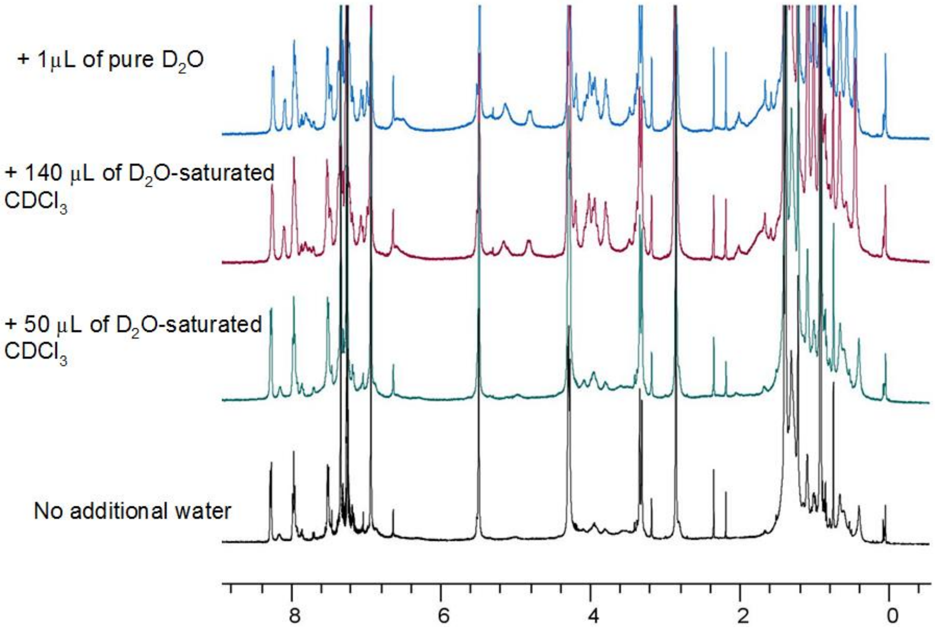

Figure S9. ^1H NMR of $[Cu^I(calix[6]tmpa)](OTf)$ ($CDCl_3$; 500MHz) at 263 K before and after addition of 50, 140 µL of D_2O-saturated $CDCl_3$ and 1 µL of pure D_2O.

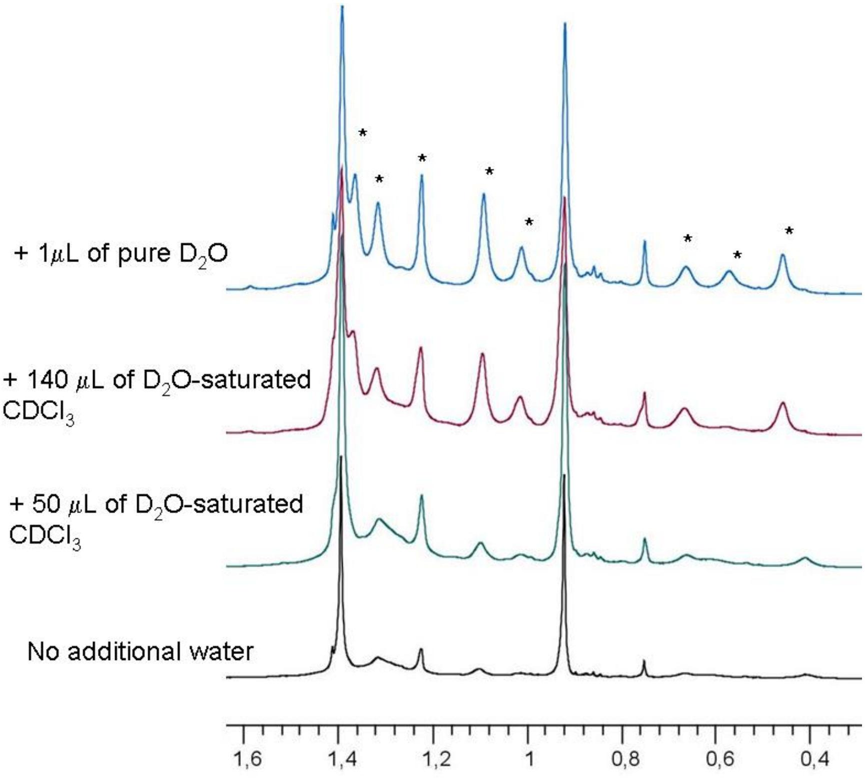

Figure S10. ^{1}H NMR spectrum of [Cu[I](calix[6]tmpa)](OTf) in the [1.6 – 0 ppm] region ($CDCl_3$; 500MHz) at 263 K before and after addition of 50, 140 µL of D_2O-saturated $CDCl_3$ and 1 µL of pure D_2O (the resonances of the growing non-C_{3v} symmetrical species are indicated with *).

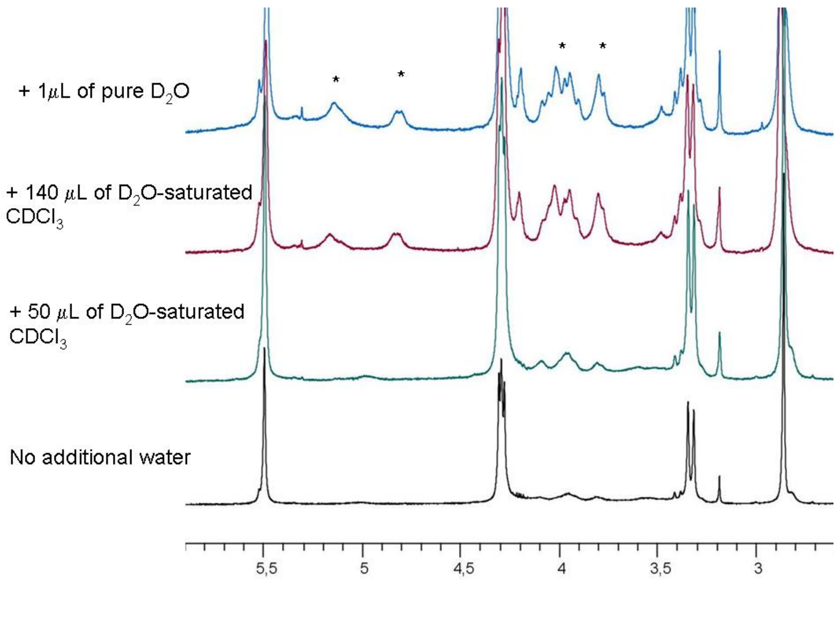

Figure S11. ^{1}H NMR spectrum of [Cu[I](calix[6]tmpa)](OTf) in the [2.5 – 6 ppm] region ($CDCl_3$; 500MHz) at 263 K before and after addition of 50, 140 µL of D_2O-saturated $CDCl_3$ and 1 µL of pure D_2O (the resonances of the growing non-C_{3v} symmetrical species are indicated with *).

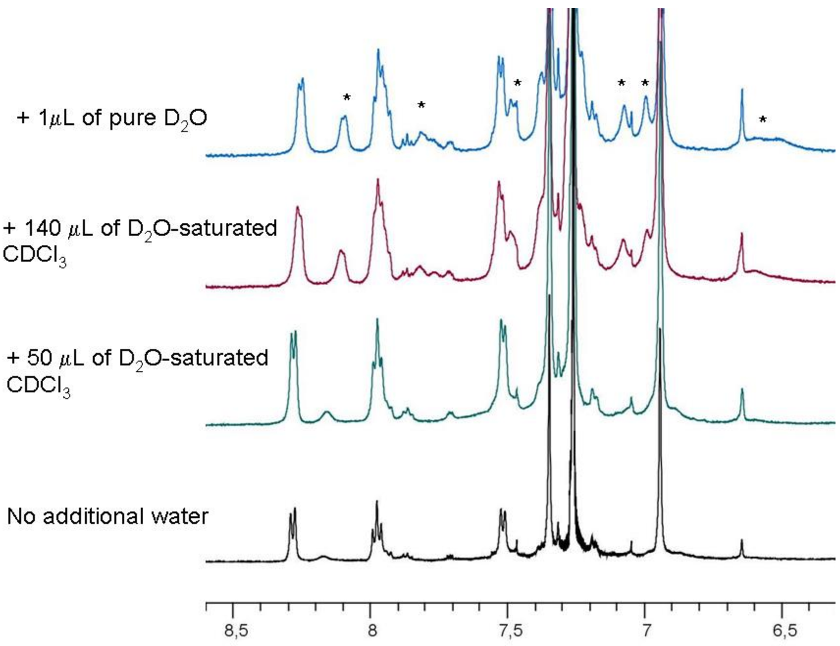

Figure S12. ^{1}H NMR spectrum of [Cu[I](calix[6]tmpa)](OTf) in the [8.5 – 6.5 ppm] region ($CDCl_3$; 500MHz) at 263 K before and after addition of 50, 140 µL of D_2O-saturated $CDCl_3$ and 1 µL of pure D_2O (the resonances of the growing non-C_{3v} symmetrical species are indicated with *).

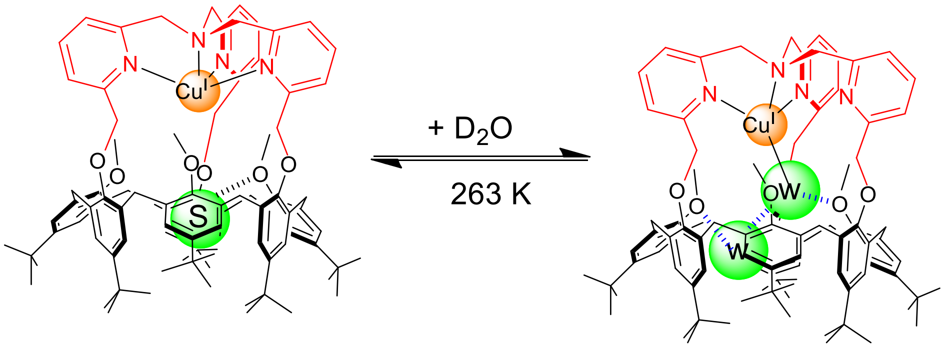

Scheme S2. Inclusion of a second molecule of water and formation of the Cu(I)-OH_2 bond with the concomitant decoordination of one pyridine. (w = D_2O, S = $CDCl_3$).

Table S3. Dynamical characterization of the $[Cu^{I}(calix[6]tmpa)(H_2O)_x]^+$ complex. The values are averaged along the DFT/MM MD trajectories. The indices refer to the anisole number and the anisoles attached to the aza-cryptant cap have even indexes while the others have odd indexes.

	$[Cu^{I}(calix[6]tmpa)(Ø)]^+$	$[Cu^{I}(calix[6]tmpa)(H_2O)]^+$		$[Cu^{I}(calix[6]tmpa)(H_2O)_2]^+$	
		CuN_3O --- N (≈65%)[a]	CuN_4 --- O (≈10%)[a]	CuN_3O --- N (≈75%)[a]	CuN_4 --- O (≈10%)[a]
d(Cu-N_2) (Å)[b]	2.14 ± 0.03	2.10 ± 0.02	2.19 ± 0.13	2.13 ± 0.02	2.19 ± 0.04
d(Cu-N_4) (Å)	2.13 ± 0.02	2.11 ± 0.02	2.16 ± 0.11	2.09 ± 0.01	2.18 ± 0.06
d(Cu-N_6) (Å)	2.22 ± 0.08	3.14 ± 0.17	2.14 ± 0.04	2.86 ± 0.013	2.17 ± 0.05
d(Cu-N_{ap}) (Å)	2.18 ± 0.01	2.25 ± 0.01	2.23 ± 0.05	2.24 ± 0.01	2.23 ± 0.02
d(Cu-O) (Å)	-	2.20 ± 0.01	2.93 ± 0.44	2.16 ± 0.01	2.52 ± 0.18
$\Sigma\phi(N_xCuN_{x'})$ (°)	351.1	-	349.1	-	345.9
d(Aro-Aro)$_{24/26/46}$ (Å)[c]	6.9/6.8/7.0	6.6/7.2/6.7		7.0/7.1/6.8	
d(C_{tBu}-C_{tBu})$_{13/15/35}$ (Å)[d]	12.8/12.9/12.4	12.7/12.7/13.0		12.5/12.6/12.5	
d(C_{tBu}-C_{tBu})$_{24/26/46}$ (Å)	6.2/6.0/5.9	5.2/5.5/7.3		5.7/5.7/6.5	
d(O_{Eth}-O_{Eth})$_{13/15/35}$ (Å)[e]	6.4/6.4/6.7	6.3/6.5/6.3		6.4/6.6/6.2	
d(O_{Eth}-O_{Eth})$_{24/26/46}$ (Å)	7.2/7.5/7.3	7.9/8.0/7.3		8.1/7.6/7.6	
$\phi(CuO_{Eth}C_{tBu})_{1/3/5}$ (°)[f]	167/160/163	157/167/165		160/161/161	
$\phi(CuO_{Eth}C_{tBu})_{2/4/6}$ (°)	115/116/111	79/117/119		97/108/114	
Simulation length (ps)	6.0	6.8		10.4	

[a]: the remaining percentage covers intermediate situations where Cu-O and Cu-N bonds are partially formed/broken. [b]: distances between copper and the pyridine nitrogen hold by anisole 2, 4 or 6. [c]: distances between the centers of masses of the anisole rings. [d]: distances between the tertiobutyl carbon atoms. [e]: distances between the ether oxygen atoms. [f]: see Fig. 4 for the definition of the angles.

Table S4. Dynamical characterization of the $[Cu^{II}(calix[6]tmpa)(H_2O)_x]^{2+}$ complex. The values are averaged along the DFT/MM MD trajectories. The indices refer to the anisole number and the anisoles attached to the aza-cryptant cap have even indexes while the others have odd indexes.

	$[Cu^{II}(calix[6]tmpa)]^{2+}$	$[Cu^{II}(calix[6]tmpa)(H_2O)]^{2+}$	$[Cu^{II}(calix[6]tmpa)(H_2O)_2]^{2+}$
d(Cu-N_2) (Å)[a]	2.06 ± 0.01	2.15 ± 0.02	2.22 ± 0.03
d(Cu-N_4) (Å)	2.08 ± 0.02	2.18 ± 0.02	2.23 ± 0.04
d(Cu-N_6) (Å)	2.07 ± 0.02	2.21 ± 0.02	2.21 ± 0.02
d(Cu-N_{ap}) (Å)	2.08 ± 0.01	2.06 ± 0.01	2.08 ± 0.01
d(Cu-O) (Å)	-	2.09 ± 0.01	2.05 ± 0.01
$\Sigma\phi(N_xCuN_{x'})$ (°)	352.5 ± 0.4	347.5 ± 0.3	347.4 ± 0.5
$\langle \tau_{TBP} \rangle = \langle \frac{\beta-\alpha}{60} \rangle$[b]	-	0.90 ± 0.01	0.89 ± 0.02
d(Aro-Aro)$_{24/26/46}$ (Å)[c]	7.0/6.9/6.7	6.6/7.2/7.1	7.2/6.9/7.1
d(C_{tBu}-C_{tBu})$_{13/15/35}$ (Å)[d]	12.5/13.3/12.7	12.6/12.1/12.5	12.3/12.6/12.4
d(C_{tBu}-C_{tBu})$_{24/26/46}$ (Å)	6.2/5.6/6.3	5.6/6.4/6.2	6.5/6.8/6.2
d(O_{Eth}-O_{Eth})$_{13/15/35}$ (Å)[e]	6.6/6.4/6.5	6.8/6.3/6.5	7.1/7.5/7.5
d(O_{Eth}-O_{Eth})$_{24/26/46}$ (Å)	7.1/7.2/7.1	7.4/7.3/7.9	6.5/6.7/6.6
$\phi(CuO_{Eth}C_{tBu})_{1/3/5}$ (°)[f]	164/157/170	156/160/159	159/157/159
$\phi(CuO_{Eth}C_{tBu})_{2/4/6}$ (°)	112/117/115	113/104/111	114/108/115
Simulation length (ps)	6.0	9.2	8.6

[a]: distances between copper and the pyridine nitrogen hold by anisole 2, 4 or 6. [b]: parameters characterizing the symmetry of the trigonal bypyramid geometry, the angles α and β are defined on Fig. S22 and S23 [c]: distances between the centers of masses of the anisole rings. [d]: distances between the tertiobutyl carbon atoms. [e]: distances between the ether oxygen atoms. [f]: see Fig. 4 for the definition of the angles.

Table S5. Probability of formation of hydrogen bonds with various lengths (d) and angle criteria (ϕ). $P_{hb}^{Hi/OCH3}$ is the probability of formation of hydrogen bond between the highest water molecule and the ether functions of the anisoles 1, 3 or 5. $P_{hb}^{Hi/OCH3}$ is the probability of formation of hydrogen bond between the lowest (second) water molecule and the ether functions of the anisoles 1, 3 or 5. $P_{hb}^{Hi/Lo}$ $P_{hb}^{Hi/OCH3}$ is the probability of formation of hydrogen bond between the two water molecules.

		$P_{hb}^{Hi/OCH3}$	$P_{hb}^{Lo/OCH3}$ **(%)**	$P_{hb}^{Hi/Lo}$ **(%)**
$[Cu^{I}(calix[6]tmpa)(H_2O)]^{+}$	d < 2.3 Å, ϕ > 140°	58.6	-	-
	d < 2.2 Å, ϕ > 140°	48.8	-	-
	d < 2.1 Å, ϕ > 150°	29.2	-	-
$[Cu^{I}(calix[6]tmpa)(H_2O)_2]^{+}$	d < 2.3 Å, ϕ > 140°	69.7	24.8	96.1
	d < 2.2 Å, ϕ > 140°	60.6	20.2	94.7
	d < 2.1 Å, ϕ > 150°	42.6	11.0	83.0
$[Cu^{II}(calix[6]tmpa)(H_2O)]^{2+}$	d < 2.3 Å, ϕ > 140°	55.5	-	-
	d < 2.2 Å, ϕ > 140°	43.6	-	-
	d < 2.1 Å, ϕ > 150°	25.4	-	-
$[Cu^{II}(calix[6]tmpa)(H_2O)_2]^{2+}$	d < 2.3 Å, ϕ > 140°	18.8	29.4	77.7
	d < 2.2 Å, ϕ > 140°	13.5	21.4	76.5
	d < 2.1 Å, ϕ > 150°	8.4	17.1	58

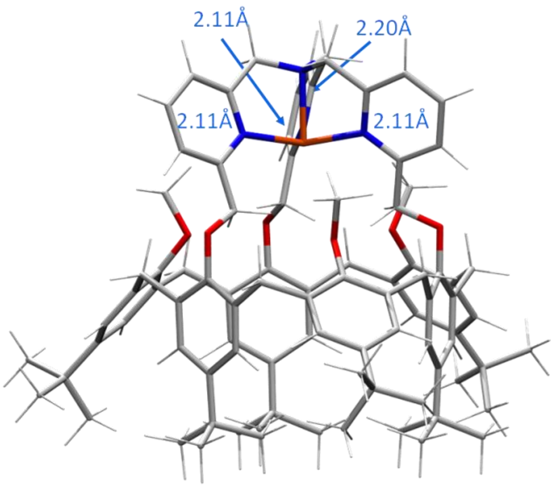

Figure S13. DFT Optimized geometries of the $[Cu^{I}(calix[6]tmpa)]^{+}$ complex

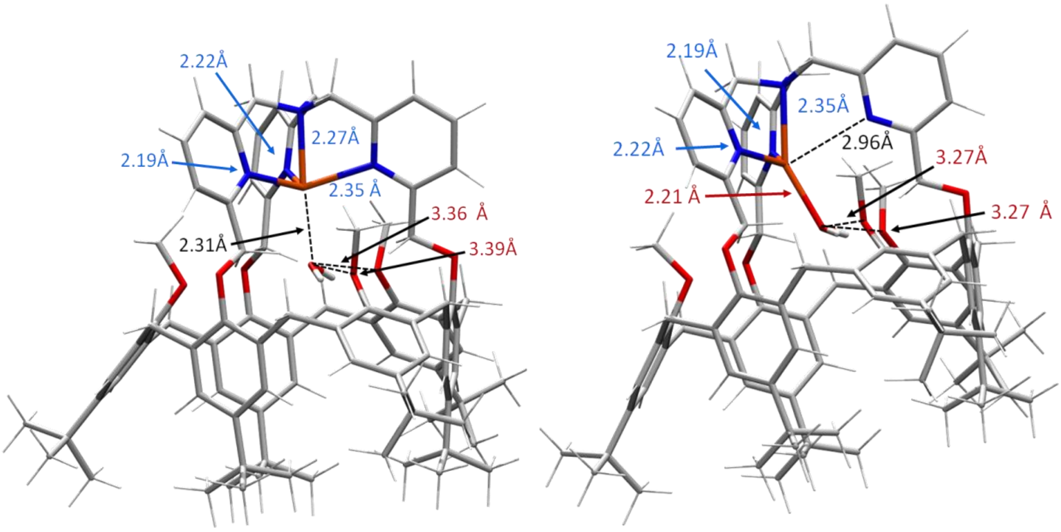

Figure S14. DFT Optimized geometries of the [Cu^{I}(calix[6]tmpa)(H_2O)]$^+$ complex (ΔE_{bind} = -41.4 kJ/mol and -43.8 kJ/mol)

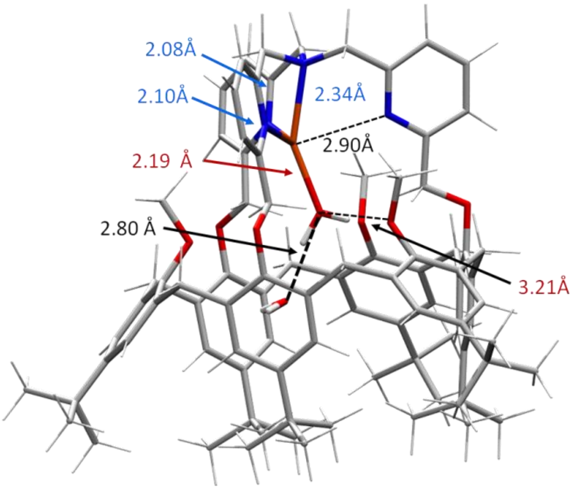

Figure S15. DFT Optimized geometries of the [Cu^{I}(calix[6]tmpa)(H_2O)$_2$]$^+$ complex (ΔE_{bind} = -102.2 kJ/mol)

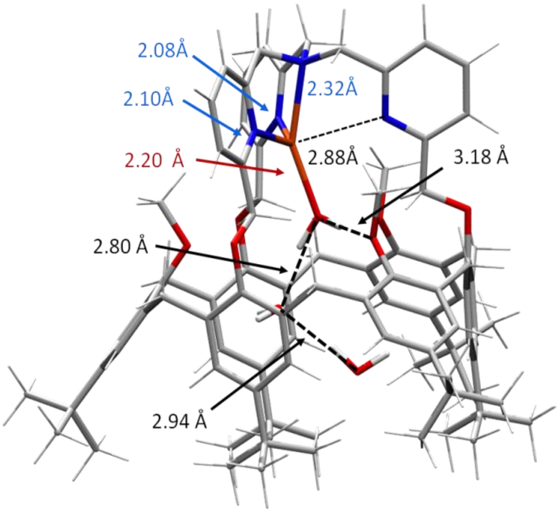

Figure S16. DFT Optimized geometries of the [Cu^{I}(calix[6]tmpa)(H_2O)$_3$]$^+$ complex (ΔE_{bind} = -141.1 kJ/mol)

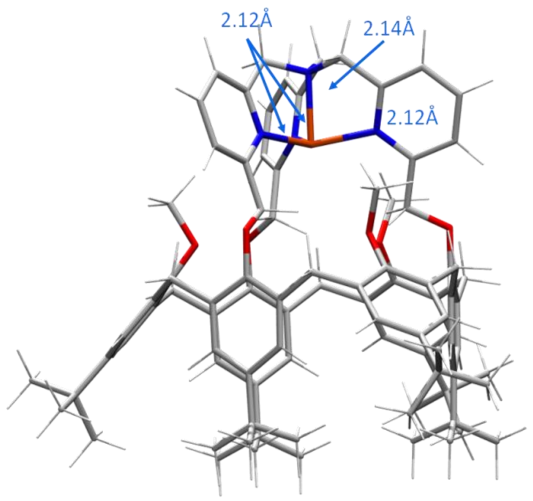

Figure S17. DFT Optimized geometries of the $[Cu^{II}(calix[6]tmpa)]^{2+}$ complex

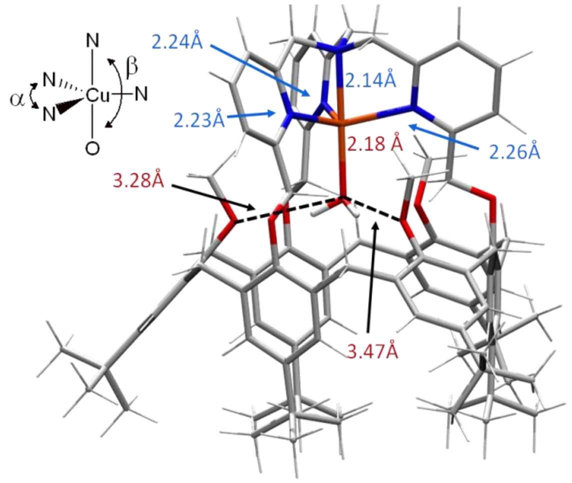

Figure S18. DFT Optimized geometries of the $[Cu^{II}(calix[6]tmpa)(H_2O)]^{2+}$ complex (ΔE_{bind} = -46.4 kJ/mol)

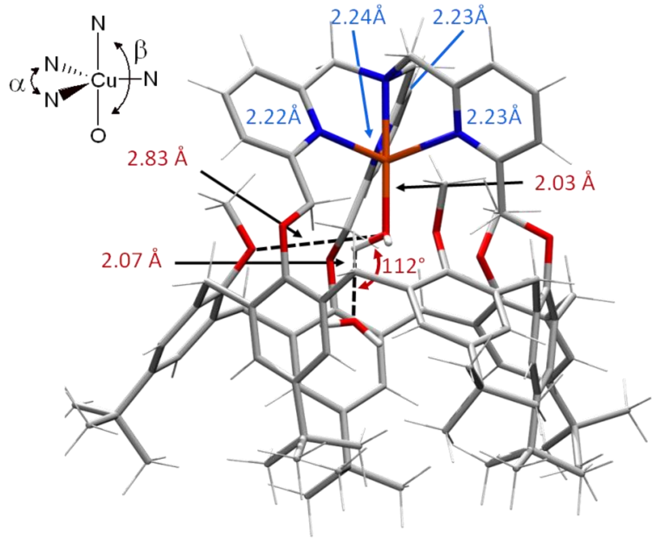

Figure S19. DFT Optimized geometries of the $[Cu^{II}(calix[6]tmpa)(H_2O)_2]^{2+}$ complex (ΔE_{bind} = -50.0 kJ/mol)

Table S6. Volume of the cavities of the complexes.

Structure	Molecule	Cavity volume (Å^3) (*esd in parentheses*)	Average volume (Å^3)
Calix[6]tmpa (apo form)	# 1	158 (10)	157 ± 15
	# 2	156 (20)	
	# 3	nd	
Cu-Calix[6]tmpa-acetonitrile	# 1	168 (10)	168 ± 20
Cu-Calix[6]tmpa-benzonitrile	# 1	190 (15)	
			187 ± 10
	# 2	184 (8)	
	# 3	186 (10)	

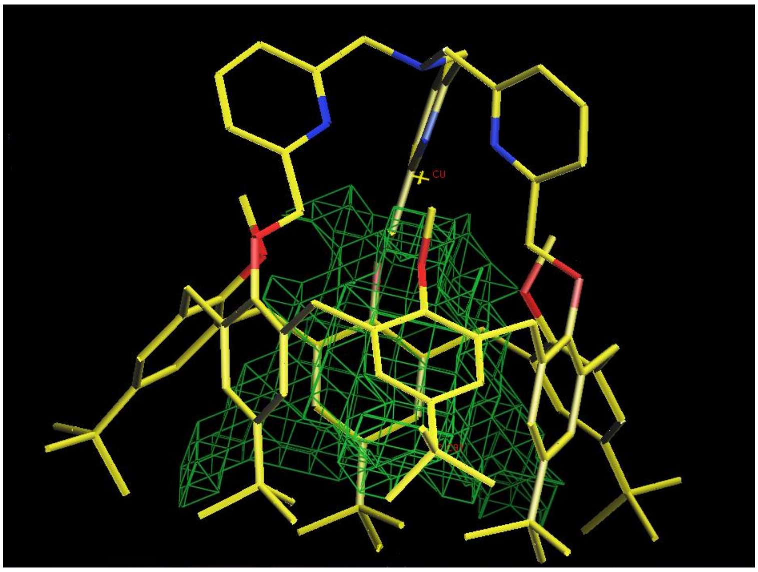

Figure S20. Delineation of the cavity in the Cu-calix[6]tmpa structure, based on Van der Waals radii: C=1.85, O=1.60, N=1.75, Cu=1.20, CH_2=1.925 and CH_3=2.00; (values in Å).

3. References and notes.

1. Z. Tyeklar, R.R. Jacobson, N. Wie, N. N. Murthy, J. Zubieta and K. D. Karlin, *J. Am. Chem. Soc.*, 1993, **115**, 2677–2689.

2. H. Nagao, N. Komeda, M. Mukaida, M. Suzuki and K. Tanaka, *Inorg. Chem.*, 1996, **35**, 6809–6815.

3. G. Izzet, X. Zeng, H. Akdas, J. Marrot and O. Reinaud, *Chem. Commun.*, 2007, 810–812.

4. The $E^0(1)$ value is approximative since a broad oxidation peak was only detected at v = 100 V s^{-1}.

5. S. Dapprich, I. Komáromi, S. K. Byun S.K., K. Morokuma and M. J. Frisch, *J. Mol. Struct. THEOCHEM* 1999, **461***,* 1–21.

6. A. M. Köster, P. Calaminici, M. E. Casida, R. Flores-Moreno, G. Geudtner, A. Goursot, T. Heine, A. Ipatov, F. Janetzko, J. M. del Campo, S. Patchkovskii, J. U. Reveles, D. R. Salahub and A. Vela, *deMon2k, The deMon developers* 2006.

7. B. R. Brooks, R. E. Bruccoleri, B. D. Olafson, D. J. States and M. Karplus, *J. Comput. Chem.*, 1983, **4**, 187–217.

8. J. P. Perdew, K. Burke and M. Ernzerhof, *Phys. Rev. Lett.*, 1996, **77***,* 3865–3868.

9. P. Calaminici, F. Janetzko, A. M. Köster, R. Mejia-Olvera and B. Zuniga-Gutierrez, *J. Chem. Phys.*, 2007, **126**, 044108.

10. Q. Wu and W. Yang, *J. Chem. Phys.*, 2002, **116**, 515–525.

11. S. J. Grimme, *J. Comput. Chem.*, 2004, **25***,* 1463–1473.

12. R Development Core Team. R: A language and environment for statistical computing. R Foundation for Statistical Computing, Vienna, Austria. 2008 ISBN 3-900051-07-0, URL http://www.R-project.org.http://www.r-project.org/

13. M. Plummer, N. Best, K. Cowles and K. Vines, 2009 coda: Output analysis and diagnostics for MCMC. R package version 0.13-4.

14. M. P. Allen and D. J. Tildesley, *Computer Simulation of Liquids*, eds Clarendon Press (Oxford, UK) 1987, 192–198.

15. A. G. Leslie, H. R. Powell, “Processing diffraction data with Mosflm" in Evolving Methods for Macromolecular Crystallography. NATO Science Series, Springer Edts. Vol. 245, 41-51.

16. The CCP4 suite of programs for protein crystallography: Collaborative Computational Project 4. *Acta Cryst.*, 1994, **D50***,* 60-763.

17. G. M. Sheldrick, *Acta Cryst*., 2008, **A64**, 112-122.

18. X. Zeng, D. Coquière, A. Alenda, E. Garrier, T. Prangé, Y. Li, O. Reinaud and I. Jabin, *Chem. Eur. J.* 2006, **12***,* 6393-6402.

19. P. T. Beurskens, G. Admiraal, W. P. Beurskens, S. Bosman, R. O. Garcia-Grande and J. M. M. Gould, C. Smykalla, Technical Report of the Crystallography Laboratory, University of Nijmegen, The Nederlands 1992.

20. A. W. Addison, T. N. Rao, J. Reedijk, J. van Rijn and G. C. Verschoor, *J. Chem. Soc., Dalton Trans.*, 1984, 1349-1356.

21. A. Nicholls, k. A. Sharp and B. Honig, P*roteins: Struc., Func and Genet.*, 1991, **11**, 281-296.

22. G. J. Kleywegt and T. A. Jones, *Acta Cryst*., 1994, **D50***,* 178-185.

23. J. Dundas, Z. Ouyang, J. Tseng, A. Binkowski, Y. Turpaz and J. Liang, *Nucl. Acids Res.* 2006, **34***,* W116-W118. (Available as a web-server at *http://sts.bioengr.uic.edu/castp/*).

24. The PyMOL Molecular Graphics System, Version 1.5.0.4 Schrödinger, LLC.
